# N_2_ Fixation in *Trichodesmium* Does Not Require Spatial Segregation from Photosynthesis

**DOI:** 10.1128/msystems.00538-22

**Published:** 2022-07-11

**Authors:** Weicheng Luo, Keisuke Inomura, Han Zhang, Ya-Wei Luo

**Affiliations:** a State Key Laboratory of Marine Environmental Science and College of Ocean and Earth Sciences, Xiamen University, Xiamen, China; b Graduate School of Oceanography, University of Rhode Island, Narragansett, Rhode Island, USA; c Marine Genomics and Biotechnology Program, Institute of Marine Science and Technology, Shandong University, Qingdao, China; University of California, Riverside

**Keywords:** *Trichodesmium*, nitrogen fixation, oxygen, temporal segregation

## Abstract

The dominant marine filamentous N_2_ fixer, *Trichodesmium*, conducts photosynthesis and N_2_ fixation during the daytime. Because N_2_ fixation is sensitive to O_2_, some previous studies suggested that spatial segregation of N_2_ fixation and photosynthesis is essential in *Trichodesmium*. However, this hypothesis conflicts with some observations where all the cells contain both photosystems and the N_2_-fixing enzyme nitrogenase. Here, we construct a systematic model simulating *Trichodesmium* metabolism, showing that the hypothetical spatial segregation is probably useless in increasing the *Trichodesmium* growth and N_2_ fixation, unless substances can efficiently transfer among cells with low loss to the environment. The model suggests that *Trichodesmium* accumulates fixed carbon in the morning and uses that in respiratory protection to reduce intracellular O_2_ during the mid-daytime, when photosynthesis is downregulated, allowing the occurrence of N_2_ fixation. A cell membrane barrier against O_2_ and alternative non-O_2_ evolving electron transfer also contribute to maintaining low intracellular O_2_. Our study provides a mechanism enabling N_2_ fixation despite the presence of photosynthesis across *Trichodesmium*.

**IMPORTANCE** The filamentous *Trichodesmium* is a globally prominent marine nitrogen fixer. A long-standing paradox is that the nitrogen-fixing enzyme nitrogenase is sensitive to oxygen, but *Trichodesmium* conducts both nitrogen fixation and oxygen-evolving photosynthesis during the daytime. Previous studies using immunoassays reported that nitrogenase was limited in some specialized *Trichodesmium* cells (termed diazocytes), suggesting the necessity of spatial segregation of nitrogen fixation and photosynthesis. However, attempts using other methods failed to find diazocytes in *Trichodesmium*, causing controversy on the existence of the spatial segregation. Here, our physiological model shows that *Trichodesmium* can maintain low intracellular O_2_ in mid-daytime and achieve feasible nitrogen fixation and growth rates even without the spatial segregation, while the hypothetical spatial segregation might not be useful if substantial loss of substances to the environment occurs when they transfer among the *Trichodesmium* cells. Our study then suggests a possible mechanism by which *Trichodesmium* can survive without the spatial segregation.

## INTRODUCTION

*Trichodesmium* sp. is a dominant contributor to marine microbial N_2_ fixation (1–3), an essential process in marine ecology and biogeochemistry. N_2_ fixation by *Trichodesmium* has been thought to be paradoxical, since it fixes N_2_ and conducts O_2_ evolving photosynthesis during the daytime (1), although nitrogenase, the enzyme catalyzing N_2_ fixation, is highly sensitive to O_2_ (4). One widely discussed hypothesis is that *Trichodesmium* may temporally segregate the two conflicting processes (5–10). Photosynthesis of *Trichodesmium* often peaks in the morning, while N_2_ fixation mainly occurs at noon or in the afternoon, when the intracellular O_2_ is low due to concurrent low photosynthesis and probably high respiration (5, 8, 9, 11, 12). This phenomenon of asynchrony in the peak timing of photosynthesis and N_2_ fixation is commonly referred to as temporal segregation of the two processes, although the two processes are not completely separated in time (5). The temporal segregation, however, is not always obvious in *Trichodesmium* (13–15), for unclear reasons.

In addition, it has been hypothesized that *Trichodesmium* segregates these two competing processes spatially (5, 7, 9, 10, 16). *Trichodesmium* exists as filamentous trichomes consisting of dozens to hundreds of cells (1), in which N_2_ fixation may be allocated in specialized cell segments (termed diazocytes) (15) and thus be spatially segregated from photosynthesis (5, 9). The spatial segregation, if it exists, requires the transfer of substances among *Trichodesmium* cells, while it is unclear how it occurs (6). As supporting evidence, some studies have revealed that nitrogenase is only distributed in diazocytes (5, 17–19). However, contradictory results have also been reported in which nitrogenase is randomly distributed in some, or even all, *Trichodesmium* cells (20–22). ^13^C and ^15^N isotope measurements via nanometer-scale secondary ion mass spectrometry (NanoSIMS) also indicate that N_2_ fixation of *Trichodesmium* might not be limited to specialized diazocytes (6). These results lead to controversy in the existence of spatial segregation between N_2_ fixation and photosynthesis in *Trichodesmium*.

Multiple mechanisms have been found or proposed to be involved in the O_2_ regulation of *Trichodesmium*. The reduced permeability of the *Trichodesmium* plasma membrane to O_2_ can slow into-cell O_2_ diffusion, which is possible considering that the membrane of Gram-negative *Trichodesmium* is surrounded by a cell envelope with multiple layers (23). A recent study also proposed that hopanoid lipid, a component of *Trichodesmium*’s membrane, may reduce the O_2_ permeability (24).

Another mechanism for O_2_ regulation is respiratory protection (RP). RP is active aerobic respiration of carbohydrates by diazotrophs to lower intracellular O_2_ to protect nitrogenase, while the produced energy is lost as heat to the environment (5, 7, 25, 26). High RP might reduce the plastoquinone pool and send negative feedback to photosystem II (PSII), further lowering intracellular O_2_ production in *Trichodesmium* (5).

Alternative electron transfer (AET), one of the photosynthetic electron transfer (PET) pathways, might also contribute to maintaining low intracellular O_2_ (8, 26). Electrons produced from PSII via the decomposition of H_2_O transfer to ferredoxin (Fd) and terminally return to H_2_O by, e.g., the Mehler reaction, forming (pseudo)cyclic electron flows around photosystem I (PSI) and resulting in zero net O_2_ production (27, 28). AET produces intracellular energy (ATP) but not the reducing agent NADPH (29). In marine diazotrophs, AET can be a complement to linear PET (LPET), in which ATP and NADPH are produced at a molar ratio (1.3:1) that is substantially lower than that (3:1) required by N_2_ fixation (27, 28, 30–32). AET can therefore benefit N_2_ fixation by providing ATP to energetically expensive N_2_ fixation while not generating O_2_ (8, 26, 33, 34).

Recently, the segregation of *Trichodesmium* N_2_ fixation and photosynthesis has been studied using a physiological cell model (7). The model consists of coarse-grained metabolic fluxes resolving key metabolisms, such as N_2_ fixation, respiration, and biomass synthesis, suggesting that combined mechanisms are essential in regulating intracellular O_2_, including RP, low permeability of cell membranes, and temporal and spatial segregations of N_2_ fixation and photosynthesis. However, the model predefined the temporal segregation of N_2_ fixation and photosynthesis and thus did not test the possibility of *Trichodesmium*’s survival without spatial segregation. Exploring such a possibility is critical in reconciling conflicting observations in which nitrogenase is found in a small group of cells (5, 17–19) or all the cells (20–22).

In the present study, we constructed a systematic physiological model of a single trichome of *Trichodesmium*, tracking the fluxes of carbon, nitrogen, O_2_, NADPH, and ATP through different intracellular pools and processes. An optimization method was applied to seek a model parameter set that maximizes the growth rate, which allows the model to self-organize its diurnal patterns of various physiological processes. Model experiments were conducted to quantitatively evaluate the importance and necessity of different strategies, including the temporal and spatial segregations of N_2_ fixation and photosynthesis, for regulating the intracellular O_2_ of *Trichodesmium* trichomes and accomplishing feasible N_2_ fixation. The results show that the hypothetical spatial segregation can be useful but is not mandatory.

## RESULTS

### General model framework.

**FIG 1:**
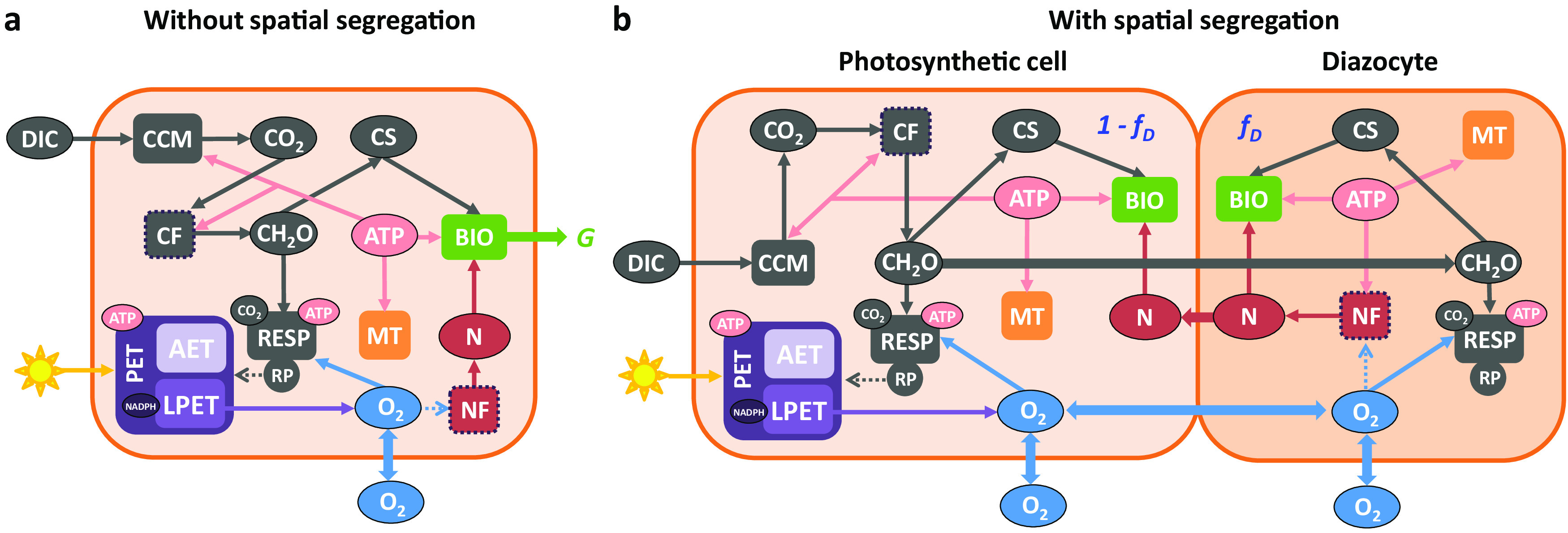
Schematic diagram of our *Trichodesmium* trichome model. N_2_ fixation and photosynthesis are not spatially segregated (a) or are spatially segregated (b). The model simulates the amount of biomass synthesized over a diel cycle and calculates a growth rate (*G*). In the model with spatial segregation (b), N_2_ is fixed in diazocytes that consist of a fraction (*f_D_*) of total cells, PET and carbon fixation occur in the remaining photosynthetic cells, and fixed carbohydrate produced in photosynthetic cells and fixed N produced in diazocytes are instantaneously transferred among the cells. For a clearer plot, arrows representing the production of ATP (by AET and LPET) and NADPH (by LPET), the consumption of NADPH (by both carbon and N_2_ fixations [dashed frames]), and the transfer of ATP and NADPH from photosynthetic cells to diazocytes are omitted. ATP produced by RP is wasted and not counted. Refer to the text for detailed descriptions. Dark orange frame, cell membrane; oval blobs, biochemical pools; rectangles, metabolic processes; solid arrows, mass or energy fluxes; dashed arrows, inhibition effects of RP on PET (gray) or of O_2_ on N_2_ fixation (blue). CCM, CO_2_-concentrating mechanism; CF, carbon fixation; NF, N_2_ fixation; RP, respiratory protection; CH_2_O, carbohydrates; CS, carbon skeleton; N, fixed nitrogen; MT, maintenance; BIO, biosynthesis.

The model (Fig. 1) estimates the growth of *Trichodesmium* trichome by simulating 12-h diurnal cycles of major intracellular processes involved in synthesizing organic carbon and fixing N_2_. The production and the consequent allocation of intracellular ATP and/or NADPH partly determine the rates of these processes. The rate of N_2_ fixation is also impacted by the temporal evolution of the intracellular O_2_ level. The modeled O_2_ inhibition on N_2_ fixation rate uses a Michaelis-Menten equation (35), in which the half-saturation coefficient ($k_{O_{2}}^{\mathrm{NF}}$= 10^−2^ mol O_2_ m^−3^) is determined by fitting a modeled gross fixed C-to-N ratio of 49:1 with the observed value of 47:1 from an experiment with *Trichodesmium* (6) (see further discussion and sensitivity tests of $k_{O_{2}}^{\mathrm{NF}}$ in the Discussion, below). The assimilated organic carbon and nitrogen accumulated at the end of the diurnal cycle are used to calculate a daily integrated growth rate. The spatial segregation of N_2_ fixation and photosynthesis into segments of model trichome is also tested. Here, we briefly introduce our model framework, while more details are described in Materials and Methods.

We first introduce our model case in which photosynthesis and N_2_ fixation are not spatially segregated (Fig. 1a). The model runs with diurnal variable light, which drives PET pathways, including LPET and AET. LPET produces O_2_, ATP, and NADPH, while AET only produces ATP (28). The ratio of LPET and AET is dynamically adjusted to fulfill the relative requirements of ATP and NADPH by all the processes. The sole N source of the model, N_2_ fixation, consumes ATP and NADPH (31, 32). CO_2_ and HCO_3_^−^ are taken up (ATP is needed in HCO_3_^−^ uptake) (36) and then fixed to carbohydrates by consuming ATP and NADPH. Some carbohydrates are further synthesized to form carbon skeletons (37). ATP is also needed for cell maintenance (38). Our model prioritizes using ATP and NADPH for N_2_ fixation over other processes, but N_2_ fixation can only proceed under low intracellular O_2_ levels (4).

The model implements RP by actively respiring carbohydrates to reduce the intracellular O_2_ while wasting the produced ATP (5, 7, 25, 26). RP, as discussed above, inhibits PET (5) and consequently slows O_2_ production. This intracellular production and consumption of O_2_, as well as the cross-cell exchange of O_2_, generate a dynamic level of intracellular O_2_ and largely regulate the sometimes-observed diurnal patterns of N_2_ fixation. Note that ATP and NADPH in the model are solely produced by LPET and AET during the daytime (39) and are instantaneously used (i.e., not stored). At the end of the daytime, the model calculates the amount of accumulated fixed carbon that needs to be respired to produce ATP, which subsequently supports maximal biosynthesis from the remaining fixed carbon and nitrogen.

The model case with spatially segregated photosynthesis and N_2_ fixation is constructed by modifying the model without the spatial segregation, to separate the trichome into N_2_-fixing (diazocytes) and photosynthetic cells (Fig. 1b). N_2_ fixation is confined in diazocytes, which are set to make up 15% of total cells (2, 16), while LPET, AET, and carbon fixation only occur in the remaining photosynthetic cells. All the materials except O_2_ are assumed to instantaneously and 100% efficiently transfer between diazocytes and photosynthetic cells and distribute evenly along the trichome (6), which is a best-case assumption for the growth of *Trichodesmium* with spatial segregation. Further evaluation and model experiments about this assumption are discussed later. Intracellular O_2_ in diazocytes and in photosynthetic cells is simulated separately. A mixed layer of O_2_ (see Fig. S1 in the supplemental material) is considered to form around the surface of the whole trichome, and the O_2_ exchange among the mixed layer, the diazocytes and the photosynthetic cells, is calculated separately, following a scheme from Staal et al. (40).

10.1128/msystems.00538-22.4 FIG S1 Schematic diagram of O_2_ diffusion modified from Staal et al. (15) under the spatial segregation. The peach space is the cytoplasm, the dark orange part is the cell membrane, the dark red part is the mixed layer, and the white part inside the dashed circle is the boundary layer. *R* is the radius of the cytoplasm, *L_g_* is the thickness of the cell membrane, *L_m_* is the thickness of the mixed layer, and *L_b_* is the thickness of the boundary layer. Download FIG S1, PDF file, 0.07 MB. Copyright © 2022 Luo et al. 2022 Luo et al. https://creativecommons.org/licenses/by/4.0/ This content is distributed under the terms of the Creative Commons Attribution 4.0 International license.

### Growth rate and daily integrated carbon and N_2_ fixation rates.

By optimizing model parameters, the model in which *Trichodesmium* trichome is not spatially segregated to diazocytes and photosynthetic cells achieves a maximal growth rate of 0.25 day^−1^ (Table 1). The modeled growth rate falls within the general observed levels of *Trichodesmium* (0.1 to 0.4 day^−1^) (13, 41–45). The modeled daily integrated fixed N (0.05 mol N per mol C per day) is coupled with the growth rate, using the Redfield ratio (46).

**TABLE 1:** Modeled growth and daily integrated carbon and N_2_ fixation rates

Model case	Growth rate (days^−1^)	Carbon fixation rate (mol C [mol C]^−1^ day^−1^)	N_2_ fixation rate (mol N [mol C]^−1^ day^−1^)
Without spatial segregation	0.25	2.24	0.05
With spatial segregation	0.51	2.21	0.11

After incorporating the spatial segregation into the model, it reaches a much higher rate of 0.51 day^−1^, which is mainly attributed to the elevated daily integrated N_2_ fixation rate (Table 1). However, the daily integrated carbon (carbohydrate) fixation rate is nearly the same in the two model cases (Table 1), indicating that much more fixed carbon is respired or wasted in the model without spatial segregation. The high growth and N_2_ fixation rates in the model without spatial segregation will be discussed later.

Nevertheless, *Trichodesmium* that does not spatially segregate photosynthesis and N_2_ fixation can still grow mainly, because it fixes a large amount of carbon in the early period and then uses that carbon in RP during the mid-day, resulting in more O_2_ consumption than is produced by photosynthesis and creating a low-O_2_ window for N_2_ fixation (further details are provided in the Discussion section, below).

### Temporal segregation of carbon and N_2_ fixations.

**FIG 2:**
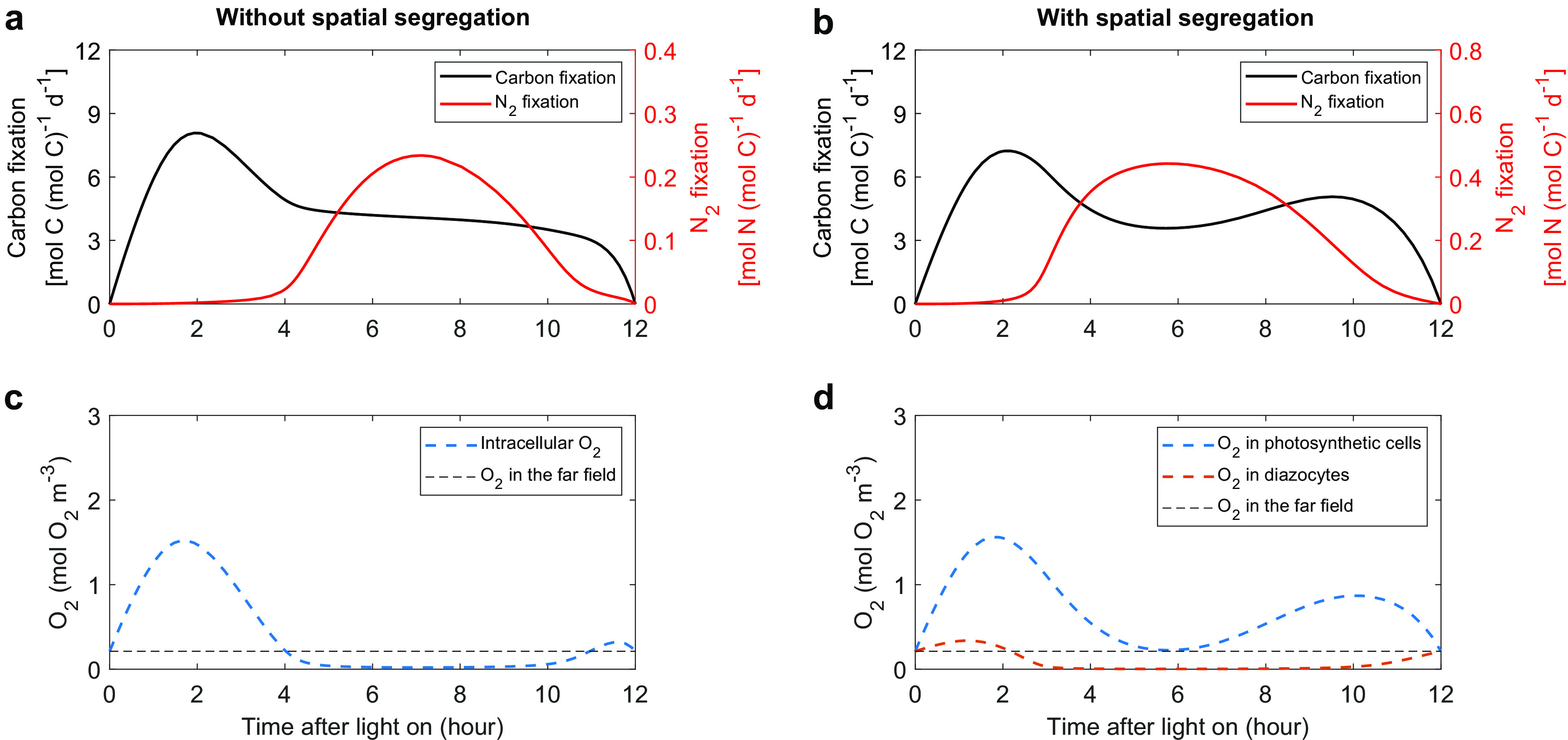
Simulated rates of C and N_2_ fixations and O_2_ concentrations. The model runs both without (a and c) and with (b and d) a spatial segregation between carbon and N_2_ fixation. The thin black dashed lines in panels c and d represent the ambient far-field O_2_ concentrations set by the model.

The simulated carbon and N_2_ fixations segregate temporally in both models with and without spatial segregation (Fig. 2a and b). These optimized results represent the patterns via which the model can reach the maximal growth rate and tentatively support the necessity of the temporal segregation between photosynthesis and N_2_ fixation in *Trichodesmium*, more of which will be discussed later. The carbon fixation rate increases to its daily peak in the first 2 h, gradually decreases to approximately half of its maximum until noon, remains nearly constant (Fig. 2a) or increases to a second peak (Fig. 2b) for another 4 h, and then reduces to 0 at the end of the light period. N_2_ fixation mainly occurs in the middle light period, when the carbon fixation rate is downregulated and a window of low intracellular O_2_ emerges (Fig. 2c and d). Compared to the model without spatial segregation, the model spatially segregating photosynthesis and N_2_ fixation has a wider low-O_2_ window in diazocytes and a longer period of N_2_ fixation (Fig. 2).

Our model generates a diurnal pattern of N_2_ fixation with a single peak, which is consistent with most previous studies of single trichomes of *Trichodesmium* (5, 6, 8, 13, 39, 41, 44, 47). N_2_ fixation in our model peaks during the later light period, which was observed in some of above studies (6, 13, 41, 47), although the exact peaking time of N_2_ fixation varied substantially, probably due to different culture and physiological conditions.

### Dynamic changes of O_2_ fluxes.

**FIG 3:**
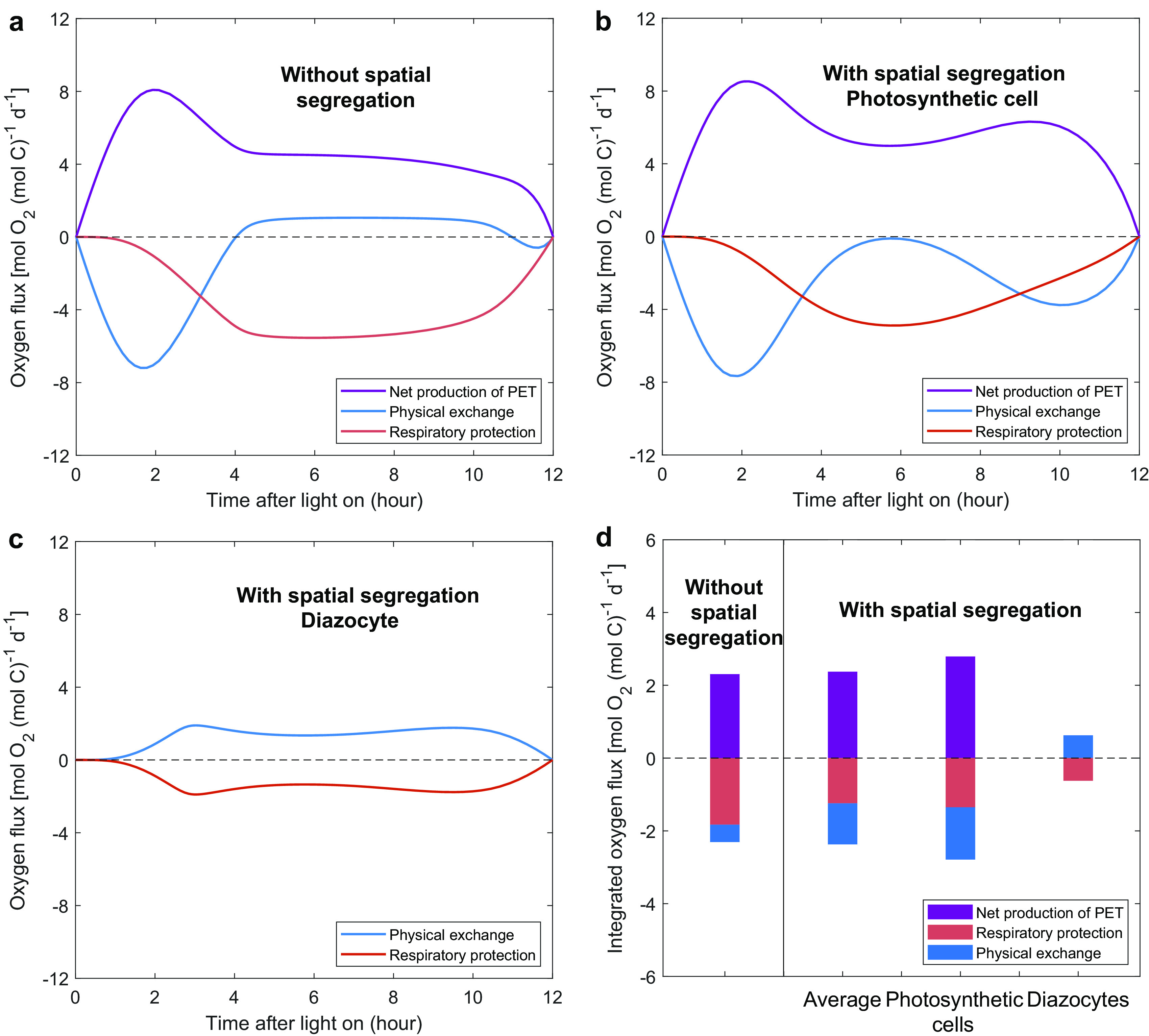
Modeled intracellular O_2_ fluxes. (a to c) Photosynthesis and N_2_ fixation are either not spatially segregated (a) or spatially segregated to photosynthetic cells (b) and diazocytes (c). (d) Daily integrated O_2_ fluxes in models with and without spatial segregation, with the results for the photosynthetic cells and diazocytes in the model with spatial segregation also shown. Positive and negative values represent gain and loss of intracellular O_2_, respectively. O_2_ fluxes in the photosynthetic cells and diazocytes (b to d) are normalized to their own respective biomass.

We first compared the intracellular O_2_ budgets in the model without spatial segregation to those in the photosynthetic cells of the model with spatial segregation (Fig. 3a and b). In the early morning (0 to 4 h), a large amount of O_2_ was quickly produced from photosynthesis, which is consistent with an observation that the gross O_2_ evolution of *Trichodesmium* was high in the late morning or midday (48). The produced O_2_ then either diffuses to the ambient environment or is respired in both cases. After that period, when O_2_ production is moderate and N_2_ fixation increases rapidly, the RP dominates the removal of intracellular O_2_ in both cases. The low intracellular O_2_ in turn leads to a physical influx of O_2_ in the model without spatial segregation (Fig. 3a). Without N_2_ fixation, the photosynthetic cells of the model with spatial segregation, however, allow a lower RP than that without spatial segregation, and meanwhile an intracellular O_2_ concentration always higher than the extracellular level causes a continuous outflux of O_2_ (Figs. 2d and 3b). For the diazocytes of the model with spatial segregation, no O_2_ is produced inside, and there is only a relatively small influx of O_2_ due to the small area of the interface (7); consequently, a low RP is enough to create a low-O_2_ window in these cells (Figs. 2d and 3c).

In terms of the daily integrated O_2_ budget, both the O_2_ consumed by RP and its ratio to photosynthetic O_2_ production in the model without spatial segregation are higher than those with spatial segregation (Fig. 3d). This is mainly because of the lowered RP requirement in both diazocytes and photosynthetic cells of the model without spatial segregation. Furthermore, with a higher intracellular O_2_, the photosynthetic cells in the model with spatial segregation can diffuse a much larger amount of O_2_ than that without spatial segregation (Figs. 2c and d and Fig. 3d).

### Carbon, ATP, and NADPH allocation.

**FIG 4:**
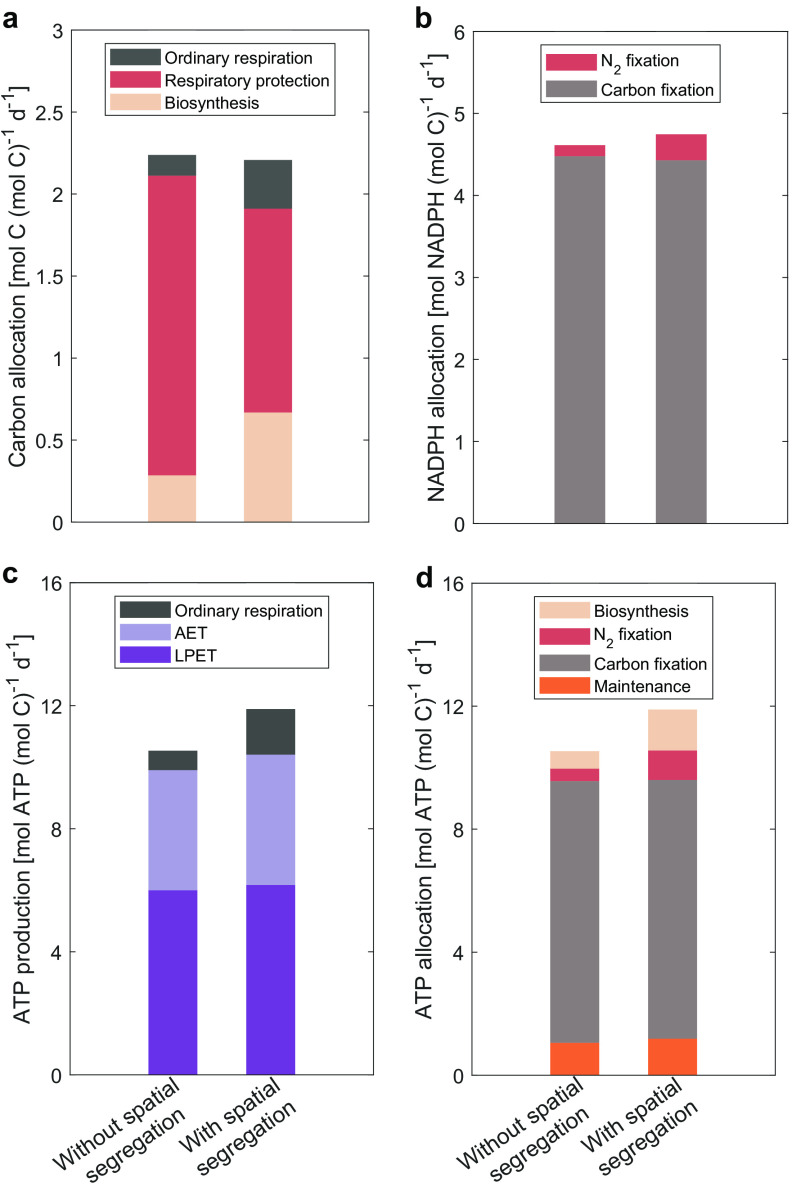
Modeled daily integrated carbon, NADPH, and ATP fluxes. Gross fixed carbon (a) and NADPH (b) allocation and ATP production (c) and allocation (d) were integrated over a diel cycle in both models, without and with spatial segregation. Note that the ordinary respiration of fixed carbon is calculated at the end of the daytime for the amount of ATP needed to synthesize biomass during the night (see text for details).

Mainly owing to the much higher fraction of gross fixed carbon consumed by RP, much less (13%) fixed carbon is synthesized to biomass in the model without spatial segregation than that with spatial segregation (30%) (Fig. 4a). To supply ATP for biosynthesis at night, more fixed carbon is respired in the model with spatial segregation than that without spatial segregation because of the higher growth in the former (Table 1 and Fig. 4a). Compared to the model without spatial segregation, ATP production is higher in the model with spatial segregation, mainly because it is inhibited less by lower RP, with slightly more ATP produced by LEPT than by AET in both cases (Fig. 4c). Hence, the model with spatial segregation is capable of supporting higher energy consumption than that without spatial segregation (Fig. 4d). In both cases, most ATP (81% and 71% in models without and with spatial segregation, respectively) is consumed by carbon fixation, while much less ATP (4% and 8% in models without and with spatial segregation, respectively) is allocated to N_2_ fixation (Fig. 4d). The fraction of NADPH allocated to carbon fixation is even higher (97% and 93% in models without and with spatial segregation, respectively), with the remaining <10% of NADPH used by N_2_ fixation, reflecting that carbon fixation requires a higher ratio of NADPH:ATP than N_2_ fixation (Fig. 4b).

## DISCUSSION

### Formation of the temporal segregation.

**FIG 5:**
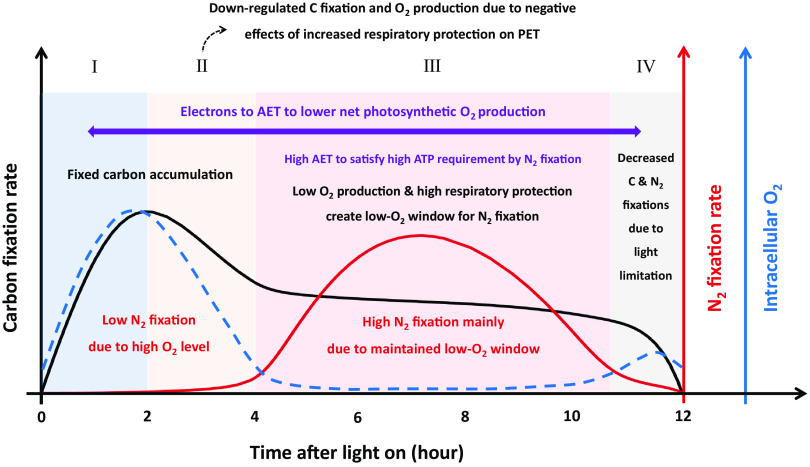
Schematic diagram illustrating the temporal segregation between C and N_2_ fixations in the model without spatial segregation. The modeled rhythms can be divided into four stages (I to IV). Black solid line, rate of carbon fixation; red solid line, rate of N_2_ fixation; blue dashed line, intracellular O_2_.

Without representing the spatial segregation between photosynthesis and N_2_ fixation in *Trichodesmium*, our model generates rhythms of carbon and N_2_ fixations (Fig. 2) that are basically consistent with sometimes-observed rhythms (6). The modeled rhythms can be divided into four stages (Fig. 5).

In the first stage (hours 0 to 2), carbon is quickly fixed and accumulates, while N_2_ is barely fixed due to high intracellular O_2_ (see Fig. S2 in the supplemental material), resulting in an increasing ratio of particulate organic C to N, a phenomenon also found in culture experiments (49).

10.1128/msystems.00538-22.5 FIG S2 Simulated concentrations of carbohydrates (black solid line), carbon skeletons (black dashed line), and fixed N (red solid line) during the light period (0 to 12 h) in the model without spatial segregation. Download FIG S2, PDF file, 0.1 MB. Copyright © 2022 Luo et al. 2022 Luo et al. https://creativecommons.org/licenses/by/4.0/ This content is distributed under the terms of the Creative Commons Attribution 4.0 International license.

In the second stage (hours 2 to 4), the accumulation of carbon skeletons (see Fig. S2) increases the cellular demand for N_2_ fixation, which in turn triggers RP (Fig. 3a). The elevated RP not only consumes more O_2_ but also partly inhibits PET and O_2_ production (Fig. 3a). These two effects, together with the diffusion of O_2_ out of the cells, quickly reduce intracellular O_2_ to a level lower than that in the environment (Fig. 2c).

In the third stage (hours 4 to 10.5), the majority of N_2_ is fixed. To maintain a low intracellular O_2_ for N_2_ fixation (Fig. 2c), the RP is at its highest level (Fig. 3a) to consume not only all the O_2_ that is photosynthetically produced at a moderate level (Fig. 2a) but also the O_2_ influx from the environment. This consequently results in a net consumption of organic carbon (see Fig. S2). The results are consistent with the net O_2_ consumption observed around a period of active N_2_ fixation in a cultured *Trichodesmium* experiment (48). Therefore, adequate carbon must be fixed and stored in the first two stages before substantial N_2_ fixation occurs, which is a reason for the necessity of the temporal segregation between carbon and N_2_ fixations.

In the last stage (hours 10.5 to 12), the accumulation of fixed N (see Fig. S2) and downregulated PET because of weakened light (Fig. 3a) causes a decrease in N_2_ fixation and in turn slows down RP (Fig. 3a). There is still a small amount of carbon fixed in this last stage (Fig. 2a).

Additional model experiments without the spatial segregation (see Text S1 and Fig. S3) show that the degree of temporal segregation between photosynthesis and N_2_ fixation largely determines daily integrated O_2_ production and RP and the ratio of net carbon to N_2_ fixations. The model reaches a maximal growth rate at an intermediate level of the temporal segregation.

10.1128/msystems.00538-22.1 TEXT S1 Full model description. Download Text S1, PDF file, 0.2 MB. Copyright © 2022 Luo et al. 2022 Luo et al. https://creativecommons.org/licenses/by/4.0/ This content is distributed under the terms of the Creative Commons Attribution 4.0 International license.

10.1128/msystems.00538-22.6FIG S3Results of model experiments without spatial segregation under different maximal synthesis rates of carbon skeletons ($v_{\mathrm{CS}}^{\max}$). (a) Comparison between the optimized case and two nonoptimized examples of the model without spatial segregation. The slow, optimal, and fast carbon skeleton (CS) syntheses are controlled by using 50%, 100%, and 200% of the optimized values of the model parameter of maximal synthesis rate of carbon skeleton ($v_{\mathrm{CS}}^{\max}$). Thin dashed lines represent the peak time of carbon fixation (black) or N_2_ fixation (red). (b) Simulated daily integrated rate of respiratory protection, peak time of carbon and N_2_ fixations, daily integrated rate of O_2_ production, and C-based and N-based specific growth rates. $v_{\mathrm{CS}}^{\max}$ ranges from 20% to 200% of the optimized value. Blue angles show the optimized model results without spatial segregation. The realized specific growth rate (dashed line) is the smaller of the C-based and N-based specific growth rates. Download FIG S3, PDF file, 1.9 MB.Copyright © 2022 Luo et al.2022Luo et al.https://creativecommons.org/licenses/by/4.0/This content is distributed under the terms of the Creative Commons Attribution 4.0 International license.

In summary, efficient carbon and N_2_ fixations with dynamic regulation of intracellular O_2_ and the requirement of sufficient accumulation of organic carbon before the period of high N_2_ fixation are the two main reasons for the modeled temporal segregation between photosynthesis and N_2_ fixation of *Trichodesmium*. Our model provides a scenario in which, even without spatially segregating N_2_ fixation and photosynthesis, *Trichodesmium* can still grow at a moderate rate with the concurrence of the two processes.

Meanwhile, our model always produces the temporal segregation between N_2_ fixation and photosynthesis, although some previous studies observed no temporal segregation in single trichomes of *Trichodesmium* (13, 14). The mechanism for how *Trichodesmium* grows without temporal segregation is certainly worthy of further investigations.

### Evaluation of the impacts from spatial segregation.

Meanwhile, the spatial segregation of photosynthesis and N_2_ fixation in different cells can increase the modeled maximum growth rate by 104% (Table 1); this is mainly caused by the expanded low-O_2_ window and the elevated N_2_ fixation in diazocytes (Fig. 2) and the lowered consumption of fixed carbon in RP (Figs. 3 and 4a). This result, however, was obtained by assuming all the synthesized materials (except O_2_) can freely and efficiently transfer between diazocytes and photosynthetic cells in the model. Although direct transfer of substances among cells has been suggested for some terrestrial filamentous N_2_-fixing cyanobacteria, such as the channels found to connect cells in *Anabaena* (50, 51), such channels or other similar mechanisms have not been discovered for *Trichodesmium*. If the substances produced in certain cells of *Trichodesmium* have to be otherwise released to extracellular environment before they can be retaken by other *Trichodesmium* cells, the loss of the transferred substances to the environment would be unavoidable. By setting a lost fraction of the intercellularly transferred materials in the model with spatial segregation (see Materials and Methods), the growth rate decreases substantially, mainly caused by the loss of fixed N, and becomes even lower than that in the model without spatial segregation when the lost fraction is higher than 50% (see Fig. S4). A loss fraction lower than this level can be difficult to reach, considering the ocean environment is dynamic and other microorganisms inhabiting areas near *Trichodesmium* can also take up these substances. Our model experiments then suggest that the benefit that *Trichodesmium* can obtain from the spatial segregation is likely overwhelmed by the loss of substances during their transfer among cells.

10.1128/msystems.00538-22.7 FIG S4 Experiments on loss of intercellularly transferred materials in the model with spatial segregation. Relative change of gross C and N_2_ fixation rates, net N assimilation rate, and specific growth rate in the model with spatial segregation to those in the model without spatial segregation. Download FIG S4, PDF file, 0.1 MB. Copyright © 2022 Luo et al. 2022 Luo et al. https://creativecommons.org/licenses/by/4.0/ This content is distributed under the terms of the Creative Commons Attribution 4.0 International license.

### Intracellular O_2_ management.

*Trichodesmium* also adopts another suite of combined intracellular O_2_ management mechanisms to protect nitrogenase. Considering the analyses above, we limit our discussion in this section only to the model results without spatial segregation.

**FIG 6:**
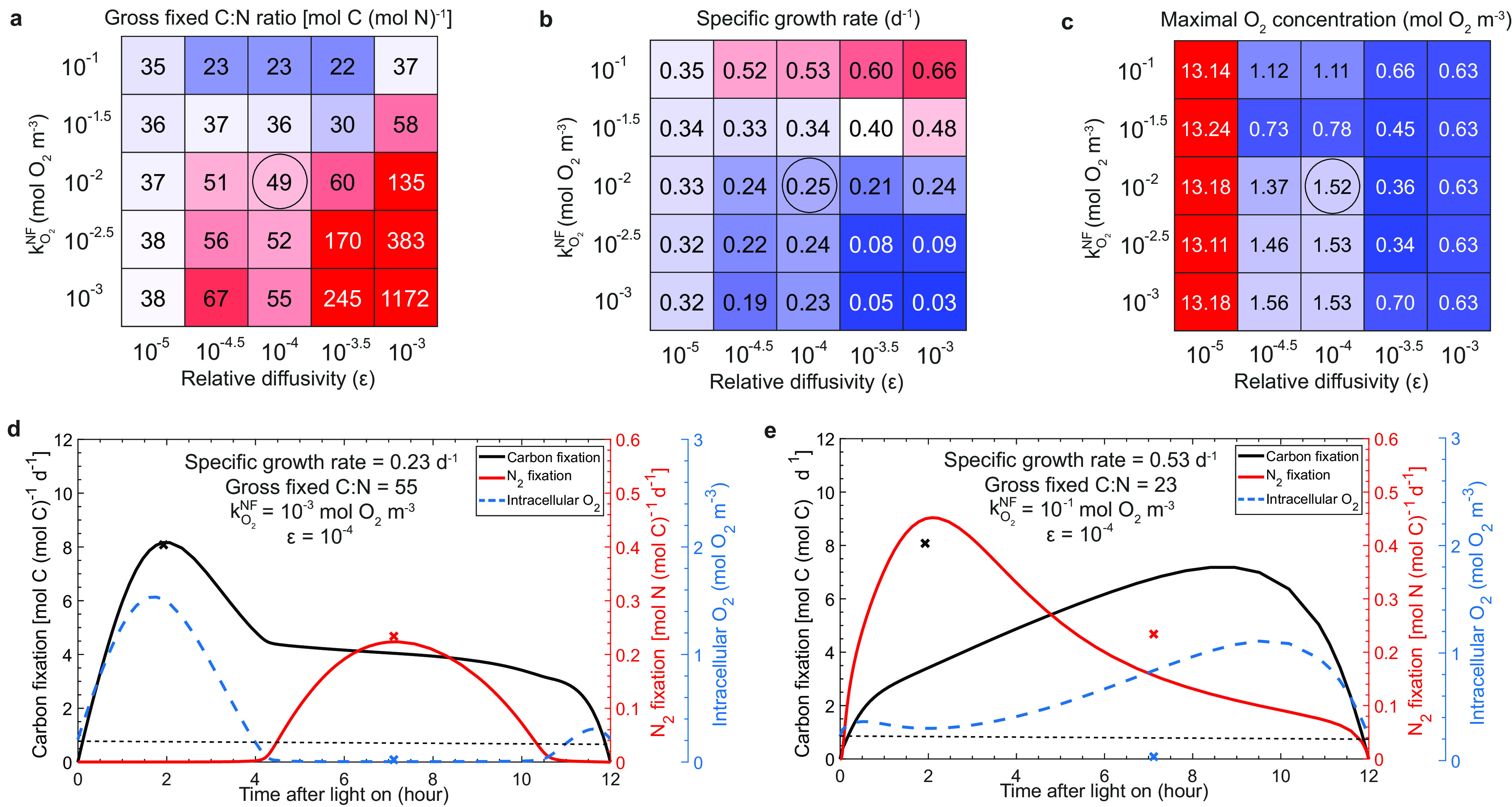
Model sensitivity tests for two model parameters related to intracellular O_2_ regulation. (a to c) The half-saturation coefficients of O_2_ inhibition on N_2_ fixation ($k_{O_{2}}^{\mathrm{NF}}$) and the relative cross-membrane O_2_ diffusivity to that in water (ε) were tested in the model without spatial segregation, showing a simulated ratio of gross C-to-N fixations (a), growth rates (b), and intracellular maximal O_2_ concentration (c). Note that values in the black circles represent the model results simulated using default values for $k_{O_{2}}^{\mathrm{NF}}$ and ε (see text). (d and e) Optimized model results without spatial segregation using a low $k_{O_{2}}^{\mathrm{NF}}$ (10^−3 ^mol O_2_ m^−3^) and a high $k_{O_{2}}^{\mathrm{NF}}$ (10^−1 ^mol O_2_ m^−3^), respectively. For comparison, several key results of the standard model without spatial segregation using a default $k_{O_{2}}^{\mathrm{NF}}$ (1 × 10^−2 ^mol O_2_ m^−3^) (i.e., Fig. 2), including the maximal carbon fixation rate (black cross), the maximal N_2_ fixation rate (red cross), and the minimal intracellular O_2_ concentration (blue cross), are marked at their corresponding occurring times in panels d and e. The thin black dashed lines in panels d and e represent the ambient far-field O_2_ concentrations predefined by the model.

Our model results suggest that proper low cell permeability to O_2_ is important to maintain the low-O_2_ window for N_2_ fixation, which is consistent with the conclusions of other studies (7, 48). The multilayer cell envelope of *Trichodesmium* makes the O_2_ diffusivity across the cell membrane thousands of times lower than that in water (7, 48, 52). Our model experiment estimates an O_2_ diffusivity of 10^−4^ of that in water (Fig. 6), a value comparable to that in another study (7). When ε is lower (10^−5^), the O_2_ produced in the early morning cannot quickly diffuse out of the cell, resulting in extremely high intracellular O_2_ concentrations (about 60 times higher than the far-field ambient O_2_ concentration) (Fig. 6c). Although not represented in our model, this high intracellular O_2_ can cause strong oxidative stress (48). When ε is higher (10^−3^), the modeled cell needs to consume much more carbon in RP, so that the modeled gross fixed C-to-N ratio was substantially increased and the modeled growth rate was greatly decreased, unless the O_2_ inhibition on N_2_ fixation was weak (i.e., high $k_{O_{2}}^{\mathrm{NF}}$) (Fig. 6a and b).

The half-saturation coefficient for the O_2_ inhibition ($k_{O_{2}}^{NF}$) (equation 3), unknown but probably substantially lower than a typical ambient O_2_ concentration (0.213 mol O_2_ m^−3^ at 34 practical salinity units (PSU) and 25°C), is estimated at 10^−2 ^mol O_2_ m^−3^. This value of $k_{O_{2}}^{NF}$, together with an ε of 10^−4^, results in a ratio of modeled gross C-to-N fixations of 49:1 (Fig. 6), which fits well to an observed value of 47:1 from an experiment of *Trichodesmium* (6). By setting a stronger O_2_ inhibition on N_2_ fixation, i.e., a lower $k_{O_{2}}^{\mathrm{NF}}$ of 10^−3 ^mol O_2_ m^−3^ (see Fig. S5), more carbon is consumed in RP, resulting in a slightly higher ratio of modeled gross fixed C to N (55:1) and a slightly lower growth rate (Fig. 6a and b), while the pattern of the temporal segregation is basically unchanged (Fig. 6d). When the O_2_ inhibition on N_2_ fixation is weaker, by setting a higher $k_{O_{2}}^{\mathrm{NF}}$ of 10^−1 ^mol O_2_ m^−3^, the model reached a much higher growth rate (0.53 day^−1^) with a lower gross fixed C-to-N ratio (23:1) (Fig. 6a and b). However, in this case, the modeled intracellular O_2_ is higher than the ambient O_2_, even when the N_2_ fixation rates are high (Fig. 6e), contradictory to our intention of this model and the common understandings that *Trichodesmium* needs to substantially reduce intracellular O_2_ to allow N_2_ fixation. The results of this experiment show that the modeled degree of temporal separation depends on the parameter $k_{O_{2}}^{\mathrm{NF}}$, which sets the strength of the O_2_ inhibition on N_2_ fixation. The model can resolve a much less pronounced temporal separation (Fig. 6e) than that in the standard model (Fig. 2a) when the O_2_ inhibition is set to be weaker, while the modeled growth rate of *Trichodesmium* is still comparable to other reported observations (41, 53).

10.1128/msystems.00538-22.8FIG S5Model experiments of O_2_ inhibition effect on N_2_ fixation. The percentage of reduced N_2_ fixation rate from its maximal rate under different intracellular O_2_ concentrations and model parameter $k_{O_{2}}^{\mathrm{NF}}$ using the Michaelis-Menten equation. Download FIG S5, PDF file, 0.1 MB.Copyright © 2022 Luo et al.2022Luo et al.https://creativecommons.org/licenses/by/4.0/This content is distributed under the terms of the Creative Commons Attribution 4.0 International license.

Upon further consideration of other reported observations showing that the gross C:N fixation ratio mainly ranges between 30 and 50 (5, 6, 13, 47), our model sensitivity tests narrowed ε and $k_{O_{2}}^{NF}$ estimates to considerably smaller ranges of ≈10^−4.5^ to 10^−4^ and 10^−2^ to 10^−1.5 ^mol O_2_ m^−3^, respectively, in which the modeled ratios of gross C and N fixations, growth rates, and intracellular levels are likely acceptable (Fig. 6).

Our model also reveals the importance of RP in regulating the intracellular O_2_ of *Trichodesmium*, in which active RP not only directly consumes O_2_ but also downregulates PET and thus photosynthetic O_2_ production (5). Further model experiments without RP showed that the much higher intracellular O_2_ levels inhibit N_2_ fixation nearly entirely (see Fig. S6). The active role of RP can also be supported by the observed strong positive correlation between the expression of nitrogenase and cytochrome oxidase (the enzyme of respiration) in *Trichodesmium* (25). The RP of *Trichodesmium* is an extra-high indirect cost of N_2_ fixation (36) and is a carbon biomass efficiency trade-off strategy commonly adopted by other marine diazotrophs (54–56).

10.1128/msystems.00538-22.9 FIG S6 Simulated rates of carbon and N_2_ fixations and O_2_ concentrations in the model experiment with no respiratory protection. Spatial segregation is not considered here. The thin black dashed line represents the ambient far-field O_2_ concentration set by the model. Download FIG S6, PDF file, 0.1 MB. Copyright © 2022 Luo et al. 2022 Luo et al. https://creativecommons.org/licenses/by/4.0/ This content is distributed under the terms of the Creative Commons Attribution 4.0 International license.

AET can be another important mechanism in N_2_-fixing *Trichodesmium*. As already discussed, AET partly satisfies the higher ATP demand by N_2_ fixation. The fraction of PET electrons passing AET is substantially higher in *Trichodesmium* (48% ± 15%) than in nondiazotrophic cyanobacteria, such as *Synechococcus* (25%) (26, 57). Even the fraction of AET in *Trichodesmium* decreases when it grows with nitrate instead of N_2_ (8). Our modeled fraction of AET is 39% on a daily basis and has a daily rhythm similar to that of N_2_ fixation (see Fig. S7b), a phenomenon also found by Milligan et al. (8). Another important role of AET in *Trichodesmium* is to scavenge O_2_ produced in PSII (27, 28) and to thus protect nitrogenase (58, 59). In our model, AET scavenges 39% of O_2_ produced in PSII, at rates comparable to those of a previous observation (26). Turning off AET in the model, the increased photosynthetic O_2_ production (by 56%) elevates RP by 11% and reduces the growth rate by 62%.

10.1128/msystems.00538-22.10FIG S7Theoretical and simulated fractions of PET electrons passing AET (*f*_AET_). (a) Evaluation of the ATP:NADPH ratio and *f*_AET_ under different ratios of instantaneous N_2_ to carbon fixation rate. For the relation between the required ATP:NADPH supply ratio and the ratio of instantaneous N_2_ to carbon fixation rate, NADPH is required for both N_2_ and carbon fixation, and ATP is calculated as the total requirement by energy-consuming processes during the daytime, including CCM, carbon fixation, N_2_ fixation, and maintenance. For the relation between *f*_AET_ and ATP: NADPH, fAET NF= 56.7% and fAET CF = 31.6% are the required values of *f*_AET_ for PET to produce ATP and NADPH at the ratios (3:1 and 1.9:1) required by N_2_ and C fixations, respectively. *f*_AET_ can also be calculated based on the ratio of instantaneous N_2_ to carbon fixation rate. (b) Simulated *f*_AET_ (black dashed line) and N_2_ fixation rates (red solid line) during the light period in the model without spatial segregation. Download FIG S7, PDF file, 0.7 MB.Copyright © 2022 Luo et al.2022Luo et al.https://creativecommons.org/licenses/by/4.0/This content is distributed under the terms of the Creative Commons Attribution 4.0 International license.

There are other possible strategies that *Trichodesmium* may use to manage intracellular O_2_, but they are not considered in our model. For instance, diazotrophs may dynamically adjust their membrane permeability to O_2_ by redistributing hopanoid lipids in the membranes to cope with instantaneous requirements (24). The high abundance of *Trichodesmium* found on sinking particles implies that remineralization of particulate organic carbon may create a low-O_2_ microenvironment for *Trichodesmium* (60). The constitution of *Trichodesmium* colonies may also protect N_2_ fixation from into-cell O_2_ diffusion by forming O_2_-depleted microzones inside the colonies (61). High respiration rates of the heterotrophic bacteria attached to *Trichodesmium* colonies (62) might also help to create a hypoxic microenvironment. However, recent studies found that no anoxia formed inside the colonies during the light period (63, 64). Nevertheless, N_2_ fixation of *Trichodesmium* colonies is often reported to be lower than that of free trichomes (48, 65, 66). How colony formation helps *Trichodesmium* manage O_2_ and impacts its N_2_ fixation, as well as its evolutionary reason, requires further research.

### Conclusions.

In this study, we constructed a physiological model of *Trichodesmium* to explore its conflict in O_2_-evolving photosynthesis and O_2_-inhibiting N_2_ fixation. Our study shows that N_2_ fixation of *Trichodesmium* is feasible without spatial separation from photosynthesis, consistent with observations in which it occurs in photosynthetic cells. Our model also suggests that the spatial segregation overall may not benefit *Trichodesmium* if substances lose during their transfer across cells. The model provides a mechanistic understanding behind the occurrence of N_2_ fixation despite the presence of photosynthesis across the trichome. Proper low cell permeability to O_2_, respiratory protection, and alternative electron transfer are key processes of *Trichodesmium* in its intracellular management to create the low-O_2_ window for N_2_ fixation. Given the diurnal changes of physiological activities simulated (e.g., photosynthetic electron transfer, carbon and nitrogen fixations), our model may be adapted in future studies to provide a further mechanistic insight regarding *Trichodesmium*, for example, into how limiting light intensity and other limiting nutrients such as iron can mediate the ATP and NAPDH production and other processes and then regulate diurnal patterns of growth and N_2_ fixation. Our model may also be used to advance our understanding of physiological processes in *Trichodesmium* colonies in their dynamic microenvironments by incorporating them into a proper physical framework.

## MATERIALS AND METHODS

In the following, we briefly describe schemes of the model without spatial segregation. The full model description, parameter values, and variables of both models without and with spatial segregation can be found in Text S1 and Tables S1 and S2 in the supplemental material.

10.1128/msystems.00538-22.2 TABLE S1 Fixed model parameters. Download Table S1, PDF file, 0.1 MB. Copyright © 2022 Luo et al. 2022 Luo et al. https://creativecommons.org/licenses/by/4.0/ This content is distributed under the terms of the Creative Commons Attribution 4.0 International license.

10.1128/msystems.00538-22.3 TABLE S2 Model variables. Download Table S2, PDF file, 0.1 MB. Copyright © 2022 Luo et al. 2022 Luo et al. https://creativecommons.org/licenses/by/4.0/ This content is distributed under the terms of the Creative Commons Attribution 4.0 International license.

### Photosynthetic pathways.

A 12-h daylight cycle is set using a sine function (67) and drives a light-dependent PET rate ($V_{\mathrm{PET}}^{I}$, in moles electrons per mole C per second), which is further inhibited by RP as already discussed:

(1) $$V_{\mathrm{PET}}\;=\;V_{\mathrm{PET}}^{I}\;\cdot \;e^{\;-\beta \;\cdot \;V_{\mathrm{RP}}}$$

where *V*_RP_ (in moles C per mole C per second) is the RP rate described later and β [in (moles C)^−1 ^ (moles C · seconds)] is a parameter for the inhibition strength.

The modeled PET is divided into LPET and AET at variable fractions. For each electron through LPET, 0.65 ATP, 0.5 NADPH, and 0.25 O_2_ are produced, while each electron through AET generates 0.65 ATP but no net NADPH or O_2_ (27). Note that this ATP production rate by AET is based on a pathway in which electrons cycle through the Mehler reaction (27), which appears to be the dominant AET pathway in *Trichodesmium* (8), although other AET pathways can have different ATP production rates (27).

N_2_ fixation and C fixation require different ratios of ATP to NADPH (3:1 and 1.9:1, respectively; see below). At each time step, after calculating the N_2_ fixation rate, the model dynamically adjusts the fraction of AET in PET (*f*_AET_), and consequently the ratio of produced ATP to NADPH, to fulfill the requirements of the N_2_ fixation rate and maximize the C fixation (see Text S1 and Fig. S7a). Therefore, our model assumes a fully efficient adjustment of fractioning of PET into LPET and AET.

### N_2_ fixation.

The N_2_ fixation, including N_2_ assimilation to NH_4_^+^ and NH_4_^+^ assimilation to glutamate, in the model consumes 9 ATP and 3 NADPH per fixed N atom (31, 32).

A possible reason that N_2_ fixation of *Trichodesmium* primarily occurs during the light period is that NADPH required by N_2_ fixation may be completely provided by PET instead of respiring carbohydrates (39). Therefore, the maximal potential that N_2_ fixation can reach [$V_{\mathrm{NF}}^{\max}$, in moles N per (moles C per second)] in our model is when NADPH and ATP produced by PET are fully allocated to N_2_ fixation:

(2) VNFmax = VPET · (1 − fAET NF) · qLPETNADPHqNFNADPH

where fAET NF = 56.7% is the required value of *f*_AET_ for PET to produce ATP and NADPH at the ratio (3:1) required by N_2_ fixation, $q_{\mathrm{LPET}}^{\mathrm{NADPH}}$ = 0.5 mol NADPH (mol electrons)^−1^ is the NADPH production quota of LPET, and $q_{\mathrm{NF}}^{\mathrm{NADPH}}$ = 3 mol NADPH (mol N)^−1^ is that required by N_2_ fixation.

N_2_ fixation in the model also depends on the carbon skeleton (CS; in moles C per mole C), fixed N (in moles N per mole C), and intracellular O_2_ (in moles O_2_ per cubic meter):

(3) $$V_{\mathrm{NF}}\;=\;V_{\mathrm{NF}}^{\max}\;\cdot \;\frac{\mathrm{CS}}{\mathrm{CS}\;+\;k_{\mathrm{CS}}^{\mathrm{NF}}}\;\cdot \;(\frac{{\text{N}}_{\max}\;-N}{{\text{N}}_{\max}})\;\cdot \;(1\;-\;\frac{{\text{O}}_{2}}{{\text{O}}_{2}+k_{{\text{O}}_{2}}^{\mathrm{NF}}\;})$$

where $k_{\mathrm{CS}}^{\mathrm{NF}}$ (in moles C per mole C) is the half-saturating coefficient of the carbon skeleton for N_2_ fixation. We assume that *Trichodesmium* tends to downregulate N_2_ fixation when the fixed N is approaching maximal N storage (N_max_, in moles N per mole C) (7). The model’s O_2_ inhibition on N_2_ fixation rate uses a Michaelis-Menten equation (35), in which the value of the half-saturation coefficient ($k_{{\text{O}}_{2}}^{\mathrm{NF}}$) for the inhibition has not been reported in the literature. Model experiments were then conducted to find and pair a $k_{{\text{O}}_{2}}^{\mathrm{NF}}$ value with another parameter, ε, as described below.

### Carbon fixation.

Each inorganic carbon (C_i_, including CO_2_ and HCO_3_^−^) is fixed into carbohydrates using 2 NADPH and 3 ATP, based on the stoichiometry of the Calvin-Benson cycle (30). Additional energy of 0.8 ATP per fixed C is used by assuming 50% C_i_ leakage, 80% C_i_ from HCO_3_^–^, and a transport cost of 0.5 ATP per HCO_3_^–^ (68, 69). As mentioned above, the rate of carbon fixation is determined with *f*_AET_ after the N_2_ fixation rate is calculated.

The carbon skeleton CS value in the model is produced from carbohydrates without energy consumption or carbon loss (37). The production rate of the carbon skeleton (*V*_CS_, in moles C per mole C per second) is stimulated by the concentration of carbohydrates (CH_2_O, in moles C per mole C), as shown using a Michaelis-Menten equation (35) and is inhibited by its own accumulation (7):

(4) $$V_{\mathrm{CS}}\;=\;v_{\mathrm{CS}}^{\max}\;\cdot \;\frac{{\mathrm{CH}}_{2}\text{O}}{{\mathrm{CH}}_{2}\mathrm{O +}k_{{\mathrm{CH}}_{2}\text{O}}^{\mathrm{CS}}}\;\cdot \;\frac{{\mathrm{CS}}_{\max}-\;\mathrm{CS}}{{\mathrm{CS}}_{\max}}$$

where $\;v_{\mathrm{CS}}^{\max}$ (in moles C per mole C per second) is the maximal production rate of the carbon skeleton, $k_{{\mathrm{CH}}_{2}\text{O}}^{\mathrm{CS}}$ (in moles C per mole C) is the half-saturation constant of carbohydrates for carbon skeleton production, and CS_max_ (in moles C per mole C) is the maximal CS storage.

### Respiratory protection.

To create a low-O_2_ environment for N_2_ fixation, high intracellular O_2_ stimulates RP. The rate of RP is also stimulated by the potential of N_2_ fixation, which is in turn elevated by light and CS and is limited by fixed N (7, 56). We then parameterized the rate of RP (in moles C per mole C per second), as follows:

(5) $$V_{\mathrm{RP}}\;=\;v_{\mathrm{RP}}^{\max}\;\cdot \;\frac{{\text{O}}_{2}}{{\text{O}}_{2}\;+\;k_{{\text{O}}_{2}}^{\mathrm{NF}}}\;\cdot \;(1\;-\;e^{\;-\;\alpha_{I}\;\cdot \;I})\;\cdot \;\frac{\mathrm{CS}}{\mathrm{CS}+\;k_{\mathrm{CS}}^{\mathrm{NF}}}\;\cdot \;(\frac{{\text{N}}_{\max}\;-\;\text{N}}{{\text{N}}_{\max}})$$

where $v_{\mathrm{RP}}^{\max}$ (in moles C per mole C per second) is the maximal RP rate and α*_I_* (per micromole per square meter per second) is the initial slope of the photosynthesis versus light curve.

### O_2_ diffusion.

The O_2_ diffusion rate between the cell cytoplasm and ambient environment (*T*_O_2__, in moles O_2_ per cubic meter per second) is simulated using a scheme from Staal et al. (40):

(6) $$T_{{\text{O}}_{2}}\;=\;\frac{-2\cdot \pi \cdot d_{{\text{O}}_{2}}\cdot L}{V}\;\cdot \;{\left\{\frac{1}{\varepsilon }\cdot \ln\left(\frac{R}{R\;+\;L_{g}}\right)\;-\;\ln\left(\frac{R\;+\;L_{g}\;+\;L_{b}}{R\;+\;L_{g}}\right)\right\}}^{-1}\;\cdot \;({\text{O}}_{2}^{E}\;-\;{\text{O}}_{2})$$

where ${\text{O}}_{2}^{E}$ is the ambient far-field O_2_ concentration set to a saturating concentration (0.213 mol O_2_ m^−3^) under typical ocean conditions of 34-PSU salinity and 25°C (70), *d*_O_2__ (in square meters per second) is the O_2_ diffusion coefficient in seawater, *L* (in meters) and *V* (in cubic meters) are the length and volume of the trichome (simplified to cylindrical geometry), respectively, ε is the ratio of the O_2_ diffusion coefficient of the cell membrane to the *d*_O_2__ and is estimated to be 10^−4^ by model experiments (described below), *R* (in meters) is the radius of the cytoplasm, *L_g_* (in meters) is the thickness of the cell membrane, and *L_b_* = 1,024 · (*R + L_g_*) is the thickness of the boundary layer (64).

### Integration of state variables during the daytime.

The temporal change rates of state variables of carbohydrates, carbon skeleton, fixed N, and intracellular O_2_ are represented in ordinary differential equations (ODEs), including all the fluxes described above. Note that NADPH and ATP are not stored but are fully consumed at each time step. Because all the rates described above have been normalized to carbon biomass, either volume, initial biomass, or the biomass concentration of *Trichodesmium* trichome does not need to be included in the model. An exception is for the ODE of intracellular O_2_ (in moles per cubic meter), in which the cellular carbon biomass concentration (*Q*_C_, which is 18,333 mol C m^−3^) (71) is used to convert carbon-normalized biological fluxes of O_2_:

(7) $$\frac{d{\text{O}}_{2}}{\mathrm{dt}}\;=\;(V_{{\text{O}}_{2}}\;-\;V_{{\text{O}}_{2}}^{\mathrm{RP}})\;\cdot \;Q_{C}\;+\;T_{{\text{O}}_{2}}$$

where $\left(V_{{\text{O}}_{2}}\;-\;V_{{\text{O}}_{2}}^{\mathrm{RP}}\right)$ is the biological production and consumption of O_2_ (by RP) in moles of O_2_ per mole C per second). These ODEs are integrated over the light period by using the MATLAB ode15s integrator (72).

### Biosynthesis and growth rate.

*Trichodesmium* might store fixed C and N during the daytime and assimilate them into biomass, mainly during the dark period (6). Therefore, for simplification, the model calculates the amount of biomass (Bio, in moles C per moles C) that can be synthesized using the carbohydrates, carbon skeletons, and fixed N at the end of the light period. Bio is the smaller of N-based (Bio_N_) and C-based biomass (Bio_C_), with Bio_N_ being calculated by dividing fixed N by the molar ratio N:C (0.159) (46). Bio_C_ is calculated from the carbohydrates and carbon skeleton, considering the mass and energy balance. The energy needed for biosynthesis is derived from the respiration of carbohydrates (${\mathrm{CH}}_{2}{\text{O}}_{\mathrm{BIO}}^{\mathrm{RESP}}$):

(8) $${\mathrm{Bio}}_{\text{C}}\;\cdot \;q_{\mathrm{BIO}}^{\mathrm{ATP}}\;\cdot \;(1\;+\;\gamma_{\mathrm{MT}})\;={\mathrm{CH}}_{2}{\text{O}}_{\mathrm{BIO}}^{\mathrm{RESP}}\;\cdot \;q_{\mathrm{RESP}}^{\mathrm{ATP}}$$

where $q_{\mathrm{BIO}}^{\mathrm{ATP}}$ (= 2 mol ATP per mol C) is the ATP requirement rate for biosynthesis (7), γ_MT_ (= 10%) represents additional energy used in maintenance, referring to all cellular processes (e.g., nutrient uptake and DNA protection) that are incalculable but require energy (38), and $q_{\mathrm{RESP}}^{\mathrm{ATP}}$ (= 5 mol ATP per mol C) is the ATP production rate from respiring carbohydrates (73). Then, the nonrespired carbohydrates and all carbon skeletons can be directly used to synthesize biomass:

(9) $${\mathrm{Bio}}_{\text{C}}\;=\;{\mathrm{CH}}_{2}O-{\mathrm{CH}}_{2}{\text{O}}_{\mathrm{BIO}}^{\mathrm{RESP}}\;+\mathrm{CS}$$

Bio_C_ and ${\mathrm{CH}}_{2}{\text{O}}_{\mathrm{BIO}}^{\mathrm{RESP}}$ are the two unknown variables in equations 8 and 9 and thus can be solved. Noting that all the rates have been normalized to carbon biomass, Bio is therefore the relative increase in biomass over 1 day. The growth rate (*G*) is then the natural log of (1 + Bio) divided by 1 day.

### Optimization of model parameters.

Our optimality-based model assumes *Trichodesmium* can regulate its intracellular processes to maximize its growth (74). In the model without spatial segregation, several important parameters, whose values are largely unknown from the literature, were optimized in large bounded ranges by using the MATLAB global optimizer MultiStart (Table 2). These optimized parameters include those related to carbon skeleton production ($v_{\mathrm{CS}}^{\max}$ and $k_{{\mathrm{CH}}_{2}\text{O}}^{\mathrm{CS}}$), N_2_ fixation ($k_{\mathrm{CS}}^{\mathrm{NF}}$), and RP ($v_{\mathrm{RP}}^{\max}$). Other parameters (see Table S1 in the supplemental material) are fixed because they are either elemental or are energy stoichiometries of metabolic activities largely constrained by known biochemical reactions, morphological parameters of *Trichodesmium*, and boundary conditions (e.g., light intensity and ambient O_2_ concentration), or they are derived from model experiments ($k_{{\text{O}}_{2}}^{\mathrm{NF}}$ and ε). To fairly compare results, for the model with spatial segregation we used the same parameter values as those for the model without spatial segregation, except for $v_{\mathrm{RP}}^{\max}$, which was reoptimized to a lower value in the model with spatial segregation (Table 2), reflecting that RP was less demanded.

**TABLE 2:** Optimized model parameters

Symbol	Units	Description	Initial range	Value after optimization
$k_{\text{CS}}^{\text{NF}}$	mol C (mol C)^−1^	Half-saturating coefficient of carbon skeleton for N_2_ fixation	[0, 1]^*a*^	0.06
$v_{\text{CS}}^{\text{max}}$	mol C (mol C)^−1^ s^−1^	Maximal production rate of carbon skeleton	[0, 5.0 × 10^−4^]^*b*^	3.7 × 10^−6^
$k_{{\text{CH}}_{\text{2}}\text{O}}^{\text{CS}}$	mol C (mol C)^−1^	Half-saturating coefficient of carbohydrate for carbon skeleton production	[0, 1]^*a*^	0.58
$v_{\text{RP}}^{\text{max}}$	mol C (mol C)^−1^ s^−1^	Maximal respiratory protection rate	[0, 5.0 × 10^−4^]^*b*^	4.5 × 10^−4^ (without spatial segregation); 4.0 × 10^−4^ (with spatial segregation)

a The upper bounds are the maximal potential of organic carbon that can be fixed over the diurnal cycle.

b The upper bounds are the maximal potential of the O_2_ production rate in photosystem II.

### Model experiments with the spatial segregation considering a cost for material transfer.

For the model with spatial segregation, given that N_2_ fixation is segregated from photosynthesis and is confined in diazocytes, the potential cost for the intercellular materials transfer was considered, including the loss of ATP, NADPH, and carbohydrate transferred into diazocytes and the loss of fixed N transfer into photosynthetic cells. To quantitively evaluate the effect of transfer cost on N_2_ fixation and growth rates, for simplicity we set the same loss fraction of transferred materials, ranging from 0% to 80%.

### Model availability.

All data generated or analyzed in this study are included in this article and its supplemental material. The code of the model in this study is available on Zenodo (https://doi.org/10.5281/zenodo.6774659).

## Supplementary Material

Reviewer comments

## References

[1] Capone DG, Zehr JP, Paerl HW, Bergman B, Carpenter EJ. 1997. *Trichodesmium*, a globally significant marine cyanobacterium. Science 276:1221–1229. doi:10.1126/science.276.5316.1221.

[2] Berman-Frank I, Lundgren P, Falkowski P. 2003. Nitrogen fixation and photosynthetic oxygen evolution in cyanobacteria. Res Microbiol 154:157–164. doi:10.1016/S0923-2508(03)00029-9. 12706503

[3] Mahaffey C, Michaels AF, Capone DG. 2005. The conundrum of marine N_2_ fixation. Am J Sci 305:546–595. doi:10.2475/ajs.305.6-8.546.

[4] Gallon JR. 1981. The oxygen sensitivity of nitrogenase: a problem for biochemists and micro-organisms. Trends Biochem Sci 6:19–23. doi:10.1016/0968-0004(81)90008-6.

[5] Berman-Frank I, Lundgren P, Chen YB, Kupper H, Kolber Z, Bergman B, Falkowski P. 2001. Segregation of nitrogen fixation and oxygenic photosynthesis in the marine cyanobacterium *Trichodesmium*. Science 294:1534–1537. doi:10.1126/science.1064082. 11711677

[6] Finzi-Hart JA, Pett-Ridge J, Weber PK, Popa R, Fallon SJ, Gunderson T, Hutcheon ID, Nealson KH, Capone DG. 2009. Fixation and fate of C and N in the cyanobacterium *Trichodesmium* using nanometer-scale secondary ion mass spectrometry. Proc Natl Acad Sci USA 106:6345–6350. doi:10.1073/pnas.0810547106. 19332780 PMC2669351

[7] Inomura K, Wilson ST, Deutsch C. 2019. Mechanistic model for the coexistence of nitrogen fixation and photosynthesis in marine Trichodesmium. mSystems 4:e00210-19. doi:10.1128/mSystems.00210-19. 31387928 PMC6687940

[8] Milligan AJ, Berman-Frank I, Gerchman Y, Dismukes GC, Falkowski PG. 2007. Light-dependent oxygen consumption in nitrogen-fixing cyanobacteria plays a key role in nitrogenase protection. J Phycol 43:845–852. doi:10.1111/j.1529-8817.2007.00395.x.

[9] Bergman B, Sandh G, Lin S, Larsson J, Carpenter EJ. 2013. *Trichodesmium*—a widespread marine cyanobacterium with unusual nitrogen fixation properties. FEMS Microbiol Rev 37:286–302. doi:10.1111/j.1574-6976.2012.00352.x. 22928644 PMC3655545

[10] Zehr JP, Capone DG. 2020. Changing perspectives in marine nitrogen fixation. Science 368:eaay9514. doi:10.1126/science.aay9514. 32409447

[11] Küpper H, Ferimazova N, Setlík I, Berman-Frank I. 2004. Traffic lights in Trichodesmium. Regulation of photosynthesis for nitrogen fixation studied by chlorophyll fluorescence kinetic microscopy. Plant Physiol 135:2120–2133. doi:10.1104/pp.104.045963. 15299119 PMC520784

[12] Subramaniam A, Carpenter EJ, Karentz D, Falkowski PG. 1999. Bio-optical properties of the marine diazotrophic cyanobacteria Trichodesmium spp. I. Absorption and photosynthetic action spectra. Limnol Oceanogr 44:608–617. doi:10.4319/lo.1999.44.3.0608.

[13] Cai X, Gao K. 2015. Levels of daily light doses under changed day-night cycles regulate temporal segregation of photosynthesis and N_2_ fixation in the cyanobacterium *Trichodesmium erythraeum* IMS101. PLoS One 10:e0135401. doi:10.1371/journal.pone.0135401. 26258473 PMC4530936

[14] Held NA, Waterbury JB, Webb EA, Kellogg RM, McIlvin MR, Jakuba M, Valois FW, Moran DM, Sutherland KM, Saito MA. 2022. Dynamic diel proteome and daytime nitrogenase activity supports buoyancy in the cyanobacterium Trichodesmium. Nat Microbiol 7:300–311. doi:10.1038/s41564-021-01028-1. 35013592 PMC10288448

[15] Paerl HW. 1994. Spatial segregation of CO_2_ fixation in *Trichodesmium* spp.: linkage to N_2_ fixation potential. J Phycol 30:790–799. doi:10.1111/j.0022-3646.1994.00790.x.

[16] El-Shehawy R, Lugomela C, Ernst A, Bergman B. 2003. Diurnal expression of *hetR* and diazocyte development in the filamentous non-heterocystous cyanobacterium *Trichodesmium erythraeum*. Microbiology (Reading) 149:1139–1146. doi:10.1099/mic.0.26170-0. 12724375

[17] Fredriksson C, Bergman B. 1997. Ultrastructural characterisation of cells specialised for nitrogen fixation in a non-heterocystous cyanobacterium, *Trichodesmium* spp. Protoplasma 197:76–85. doi:10.1007/BF01279886.

[18] Lin S, Henze S, Lundgren P, Bergman B, Carpenter EJ. 1998. Whole-cell immunolocalization of nitrogenase in marine diazotrophic cyanobacteria, *Trichodesmium* spp. Appl Environ Microbiol 64:3052–3058. doi:10.1128/AEM.64.8.3052-3058.1998. 9687472 PMC106814

[19] Sandh G, Xu LH, Bergman B. 2012. Diazocyte development in the marine diazotrophic cyanobacterium *Trichodesmium*. Microbiology (Reading) 158:345–352. doi:10.1099/mic.0.051268-0. 22053003

[20] Paerl HW, Priscu JC, Brawner DL. 1989. Immunochemical localization of nitrogenase in marine *Trichodesmium* aggregates: relationship to N_2_ fixation potential. Appl Environ Microbiol 55:2965–2975. doi:10.1128/aem.55.11.2965-2975.1989. 16348057 PMC203199

[21] Bergman B, Carpenter EJ. 1991. Nitrogenase confined to randomly distributed trichomes in the marine cyanobacterium *Trichodesmium thiebautii*. J Phycol 27:158–165. doi:10.1111/j.0022-3646.1991.00158.x.

[22] Ohki K. 2008. Intercellular localization of nitrogenase in a non-heterocystous cyanobacterium (cyanophyte), *Trichodesmium* sp. NIBB1067. J Oceanogr 64:211–216. doi:10.1007/s10872-008-0016-2.

[23] Siddiqui PJA, Carpenter EJ, Bergman B. 1992. *Trichodesmium*: ultrastructure and protein localization, p 9–28. *In* Carpenter EJ, Capone DG, Rueter JG (ed), Marine pelagic cyanobacteria: Trichodesmium and other diazotrophs. Springer, Dordrecht, the Netherlands.

[24] Cornejo-Castillo FM, Zehr JP. 2019. Hopanoid lipids may facilitate aerobic nitrogen fixation in the ocean. Proc Natl Acad Sci USA 116:18269–18271. doi:10.1073/pnas.1908165116. 31451638 PMC6744863

[25] Bergman B, Siddiqui PJA, Carpenter EJ, Peschek GA. 1993. Cytochrome oxidase: subcellular distribution and relationship to nitrogenase expression in the nonheterocystous marine cyanobacterium *Trichodesmium thiebautii*. Appl Environ Microbiol 59:3239–3244. doi:10.1128/aem.59.10.3239-3244.1993. 16349062 PMC182443

[26] Kana TM. 1993. Rapid oxygen cycling in *Trichodesmium thiebautii*. Limnol Oceanogr 38:18–24. doi:10.4319/lo.1993.38.1.0018.

[27] Geider RJ, Moore CM, Ross ON. 2009. The role of cost–benefit analysis in models of phytoplankton growth and acclimation. Plant Ecol Div 2:165–178. doi:10.1080/17550870903300949.

[28] Allen JF. 2003. Cyclic, pseudocyclic and noncyclic photophosphorylation: new links in the chain. Trends Plant Sci 8:15–19. doi:10.1016/s1360-1385(02)00006-7. 12523995

[29] Behrenfeld MJ, Halsey KH, Milligan AJ. 2008. Evolved physiological responses of phytoplankton to their integrated growth environment. Philos Trans R Soc B 363:2687–2703. doi:10.1098/rstb.2008.0019. PMC2606763 18487129

[30] Baker NR, Harbinson J, Kramer DM. 2007. Determining the limitations and regulation of photosynthetic energy transduction in leaves. Plant Cell Environ 30:1107–1125. doi:10.1111/j.1365-3040.2007.01680.x. 17661750

[31] Flores E, Herrero A. 1994. Assimilatory nitrogen metabolism and its regulation, p 487–517. *In* Bryant DA (ed), The Molecular Biology of Cyanobacteria. Kluwer Academic Publishers, Dordrecht, the Netherlands.

[32] Flores E, Frías JE, Rubio LM, Herrero A. 2005. Photosynthetic nitrate assimilation in cyanobacteria. Photosynth Res 83:117–133. doi:10.1007/s11120-004-5830-9. 16143847

[33] Behrenfeld MJ, Milligan AJ. 2013. Photophysiological expressions of iron stress in phytoplankton. Annu Rev Mar Sci 5:217–246. doi:10.1146/annurev-marine-121211-172356. 22881354

[34] Cerdan-Garcia E, Baylay A, Polyviou D, Woodward EMS, Wrightson L, Mahaffey C, Lohan MC, Moore CM, Bibby TS, Robidart JC. 2022. Transcriptional responses of *Trichodesmium* to natural inverse gradients of Fe and P availability. ISME J 16:1055–1064. doi:10.1038/s41396-021-01151-1. 34819612 PMC8941076

[35] Rogers A, Gibon Y. 2009. Enzyme kinetics: theory and practice, p 71–103. *In* Schwender J (ed), Plant Metabolic Networks. Springer, New York, NY. doi:10.1007/978-0-387-78745-9_4.

[36] Kranz SA, Eichner M, Rost B. 2011. Interactions between CCM and N_2_ fixation in *Trichodesmium*. Photosynth Res 109:73–84. doi:10.1007/s11120-010-9611-3. 21190135

[37] Rabouille S, Staal M, Stal LJ, Soetaert K. 2006. Modeling the dynamic regulation of nitrogen fixation in the cyanobacterium *Trichodesmium* sp. Appl Environ Microbiol 72:3217–3227. doi:10.1128/AEM.72.5.3217-3227.2006. 16672460 PMC1472389

[38] Luo YW, Shi D, Kranz SA, Hopkinson BM, Hong H, Shen R, Zhang F. 2019. Reduced nitrogenase efficiency dominates response of the globally important nitrogen fixer *Trichodesmium* to ocean acidification. Nat Commun 10:1521. doi:10.1038/s41467-019-09554-7. 30944323 PMC6447586

[39] Staal M, Rabouille S, Stal LJ. 2007. On the role of oxygen for nitrogen fixation in the marine cyanobacterium *Trichodesmium* sp. Environ Microbiol 9:727–736. doi:10.1111/j.1462-2920.2006.01195.x. 17298372

[40] Staal M, Meysman FJ, Stal LJ. 2003. Temperature excludes N_2_-fixing heterocystous cyanobacteria in the tropical oceans. Nature 425:504–507. doi:10.1038/nature01999. 14523445

[41] Hong H, Shen R, Zhang F, Wen Z, Chang S, Lin W, Kranz SA, Luo YW, Kao SJ, Morel FMM, Shi D. 2017. The complex effects of ocean acidification on the prominent N_2_-fixing cyanobacterium *Trichodesmium*. Science 356:527–531. doi:10.1126/science.aal2981. 28450383

[42] Shi D, Kranz SA, Kim JM, Morel FM. 2012. Ocean acidification slows nitrogen fixation and growth in the dominant diazotroph *Trichodesmium* under low-iron conditions. Proc Natl Acad Sci USA 109:E3094–E3100. doi:10.1073/pnas.1216012109. 23071328 PMC3494951

[43] Jiang H-B, Fu F-X, Rivero-Calle S, Levine NM, Sañudo-Wilhelmy SA, Qu P-P, Wang X-W, Pinedo-Gonzalez P, Zhu Z, Hutchins DA. 2018. Ocean warming alleviates iron limitation of marine nitrogen fixation. Nat Clim Chang 8:709–712. doi:10.1038/s41558-018-0216-8.

[44] Eichner M, Kranz SA, Rost B. 2014. Combined effects of different CO2 levels and N sources on the diazotrophic cyanobacterium Trichodesmium. Physiol Plant 152:316–330. doi:10.1111/ppl.12172. 24547877 PMC4260171

[45] Hutchins DA, Fu FX, Zhang Y, Warner ME, Feng Y, Portune K, Bernhardt PW, Mulholland MR. 2007. CO_2_ control of *Trichodesmium* N_2_ fixation, photosynthesis, growth rates, and elemental ratios: implications for past, present, and future ocean biogeochemistry. Limnol Oceanogr 52:1293–1304. doi:10.4319/lo.2007.52.4.1293.

[46] LaRoche J, Breitbarth E. 2005. Importance of the diazotrophs as a source of new nitrogen in the ocean. J Sea Res 53:67–91. doi:10.1016/j.seares.2004.05.005.

[47] Wannicke N, Koch BP, Voss M. 2009. Release of fixed N_2_ and C as dissolved compounds by *Trichodesmium erythreum* and *Nodularia spumigena* under the influence of high light and high nutrient (P). Aquat Microb Ecol 57:175–189. doi:10.3354/ame01343.

[48] Eichner M, Thoms S, Rost B, Mohr W, Ahmerkamp S, Ploug H, Kuypers MMM, de Beer D. 2019. N_2_ fixation in free-floating filaments of *Trichodesmium* is higher than in transiently suboxic colony microenvironments. New Phytol 222:852–863. doi:10.1111/nph.15621. 30507001 PMC6590460

[49] Kranz SA, Sültemeyer D, Richter K-U, Rost B. 2009. Carbon acquisition by *Trichodesmium*: the effect of *p*CO_2_ and diurnal changes. Limnol Oceanogr 54:548–559. doi:10.4319/lo.2009.54.2.0548.

[50] Nürnberg DJ, Mariscal V, Bornikoel J, Nieves-Morión M, Krauß N, Herrero A, Maldener I, Flores E, Mullineaux CW. 2015. Intercellular diffusion of a fluorescent sucrose analog via the septal junctions in a filamentous cyanobacterium. mBio 6. doi:10.1128/mBio.02109-14. PMC4453526 25784700

[51] Omairi-Nasser A, Haselkorn R, Austin J. 2014. Visualization of channels connecting cells in filamentous nitrogen-fixing cyanobacteria. FASEB J 28:3016–3022. doi:10.1096/fj.14-252007. 24675362

[52] MacDougall JD, McCabe M. 1967. Diffusion coefficient of oxygen through tissues. Nature 215:1173–1174. doi:10.1038/2151173a0. 6061810

[53] Zhang F, Hong H, Kranz SA, Shen R, Lin W, Shi D. 2019. Proteomic responses to ocean acidification of the marine diazotroph Trichodesmium under iron-replete and iron-limited conditions. Photosynth Res 142:17–34. doi:10.1007/s11120-019-00643-8. 31077001

[54] Nicholson DP, Stanley RHR, Doney SC. 2018. A phytoplankton model for the allocation of gross photosynthetic energy including the trade-offs of diazotrophy. J Geophys Res Biogeosci 123:1796–1816. doi:10.1029/2017JG004263.

[55] Inomura K, Bragg J, Follows MJ. 2017. A quantitative analysis of the direct and indirect costs of nitrogen fixation: a model based on *Azotobacter vinelandii*. ISME J 11:166–175. doi:10.1038/ismej.2016.97. 27740611 PMC5315487

[56] Inomura K, Deutsch C, Wilson ST, Masuda T, Lawrenz E, Lenka B, Sobotka R, Gauglitz JM, Saito MA, Prášil O, Follows MJ. 2019. Quantifying oxygen management and temperature and light dependencies of nitrogen fixation by *Crocosphaera watsonii*. mSphere 4:e00531-19. doi:10.1128/mSphere.00531-19. 31826967 PMC6908418

[57] Kana TM. 1992. Relationship between photosynthetic oxygen cycling and carbon assimilation in *Synechococcus* WH7803 (Cyanophyta). J Phycol 28:304–308. doi:10.1111/j.0022-3646.1992.00304.x.

[58] Halsey KH, Jones BM. 2015. Phytoplankton strategies for photosynthetic energy allocation. Annu Rev Mar Sci 7:265–297. doi:10.1146/annurev-marine-010814-015813. 25149563

[59] Kustka A, Saudo-Wilhelmy S, Carpenter EJ, Capone DG, Raven JA. 2003. A revised estimate of the iron use efficiency of nitrogen fixation, with special reference to the marine cyanobacterium *Trichodesmium* spp. (Cyanophyta). J Phycol 39:12–25. doi:10.1046/j.1529-8817.2003.01156.x.

[60] Farnelid H, Turk-Kubo K, Ploug H, Ossolinski JE, Collins JR, Van Mooy BAS, Zehr JP. 2019. Diverse diazotrophs are present on sinking particles in the North Pacific subtropical gyre. ISME J 13:170–182. doi:10.1038/s41396-018-0259-x. 30116043 PMC6299005

[61] Paerl HW, Bebout BM. 1988. Direct measurement of O_2_-depleted microzones in marine *Oscillatoria*: relation to N_2_ fixation. Science 241:442–445. doi:10.1126/science.241.4864.442. 17792609

[62] Frischkorn KR, Haley ST, Dyhrman ST. 2018. Coordinated gene expression between *Trichodesmium* and its microbiome over day-night cycles in the North Pacific subtropical gyre. ISME J 12:997–1007. doi:10.1038/s41396-017-0041-5. 29382945 PMC5864210

[63] Eichner MJ, Klawonn I, Wilson ST, Littmann S, Whitehouse MJ, Church MJ, Kuypers MM, Karl DM, Ploug H. 2017. Chemical microenvironments and single-cell carbon and nitrogen uptake in field-collected colonies of *Trichodesmium* under different *p*CO_2_. ISME J 11:1305–1317. doi:10.1038/ismej.2017.15. 28398346 PMC5437350

[64] Klawonn I, Eichner MJ, Wilson ST, Moradi N, Thamdrup B, Kummel S, Gehre M, Khalili A, Grossart HP, Karl DM, Ploug H. 2020. Distinct nitrogen cycling and steep chemical gradients in *Trichodesmium* colonies. ISME J 14:399–412. doi:10.1038/s41396-019-0514-9. 31636364 PMC6976679

[65] Orcutt KM, Lipschultz F, Gundersen K, Arimoto R, Michaels AF, Knap AH, Gallon JR. 2001. A seasonal study of the significance of N_2_ fixation by *Trichodesmium* spp. at the Bermuda Atlantic Time-series Study (BATS) site. Deep Sea Res II 48:1583–1608. doi:10.1016/S0967-0645(00)00157-0.

[66] Sohm JA, Subramaniam A, Gunderson TE, Carpenter EJ, Capone DG. 2011. Nitrogen fixation by *Trichodesmium* spp. and unicellular diazotrophs in the North Pacific subtropical gyre. J Geophys Res 116:G03002.

[67] Reimers AM, Knoop H, Bockmayr A, Steuer R. 2017. Cellular trade-offs and optimal resource allocation during cyanobacterial diurnal growth. Proc Natl Acad Sci USA 114:E6457–E6465. doi:10.1073/pnas.1617508114. 28720699 PMC5547584

[68] Eichner M, Thoms S, Kranz SA, Rost B. 2015. Cellular inorganic carbon fluxes in *Trichodesmium*: a combined approach using measurements and modelling. J Exp Bot 66:749–759. doi:10.1093/jxb/eru427. 25429001 PMC4321539

[69] Raven JA, Beardall J, Giordano M. 2014. Energy costs of carbon dioxide concentrating mechanisms in aquatic organisms. Photosynth Res 121:111–124. doi:10.1007/s11120-013-9962-7. 24390639

[70] Benson BB, Krause D. 1984. The concentration and isotopic fractionation of oxygen dissolved in freshwater and seawater in equilibrium with the atmosphere. Limnol Oceanogr 29:620–632. doi:10.4319/lo.1984.29.3.0620.

[71] Bratbak G, Dundas I. 1984. Bacterial dry matter content and biomass estimations. Appl Environ Microbiol 48:755–757. doi:10.1128/aem.48.4.755-757.1984. 6508285 PMC241608

[72] Shampine LF, Reichelt MW. 1997. The MATLAB ODE suite. SIAM J Sci Comput 18:1–22. doi:10.1137/S1064827594276424.

[73] Mitchell P. 1970. Aspects of the chemiosmotic hypothesis. Biochem J 116:5–6. 10.1042/bj1160005p PMC1185429 4244889

[74] Pahlow M, Dietze H, Oschlies A. 2013. Optimality-based model of phytoplankton growth and diazotrophy. Mar Ecol Prog Ser 489:1–16. doi:10.3354/meps10449.

