# Supplementary figures and images for: N2 Fixation in Trichodesmium Does Not Require Spatial Segregation from Photosynthesis
Source: mSystems. 2022 Jul 11;7(4):e00538-22. doi: 10.1128/msystems.00538-22 (PMC9426587; doi:10.1128/msystems.00538-22)

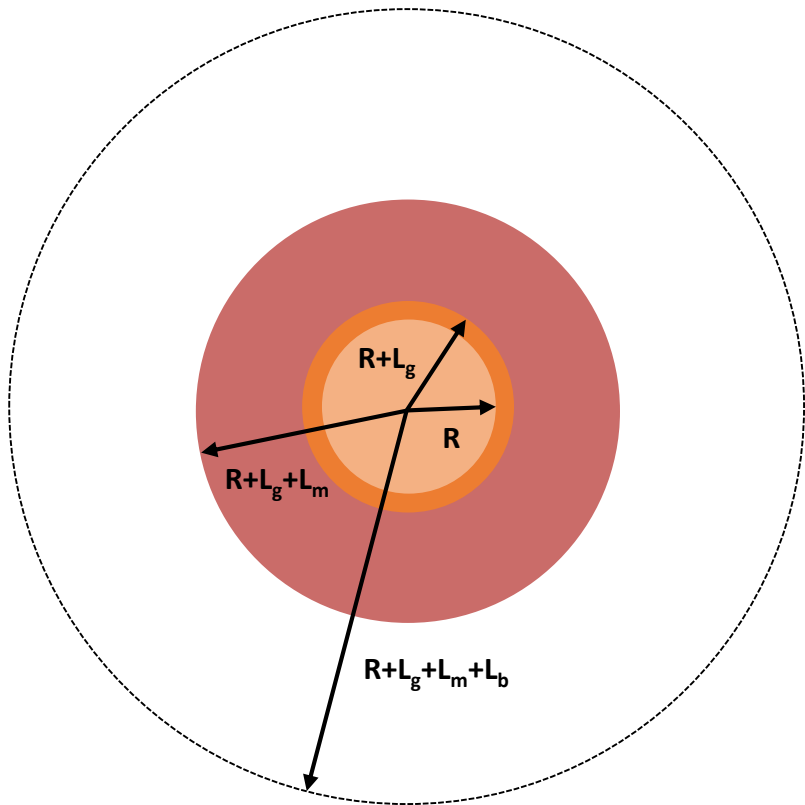

Supplement: FIG S1 [file msystems.00538-22-s0001.pdf]

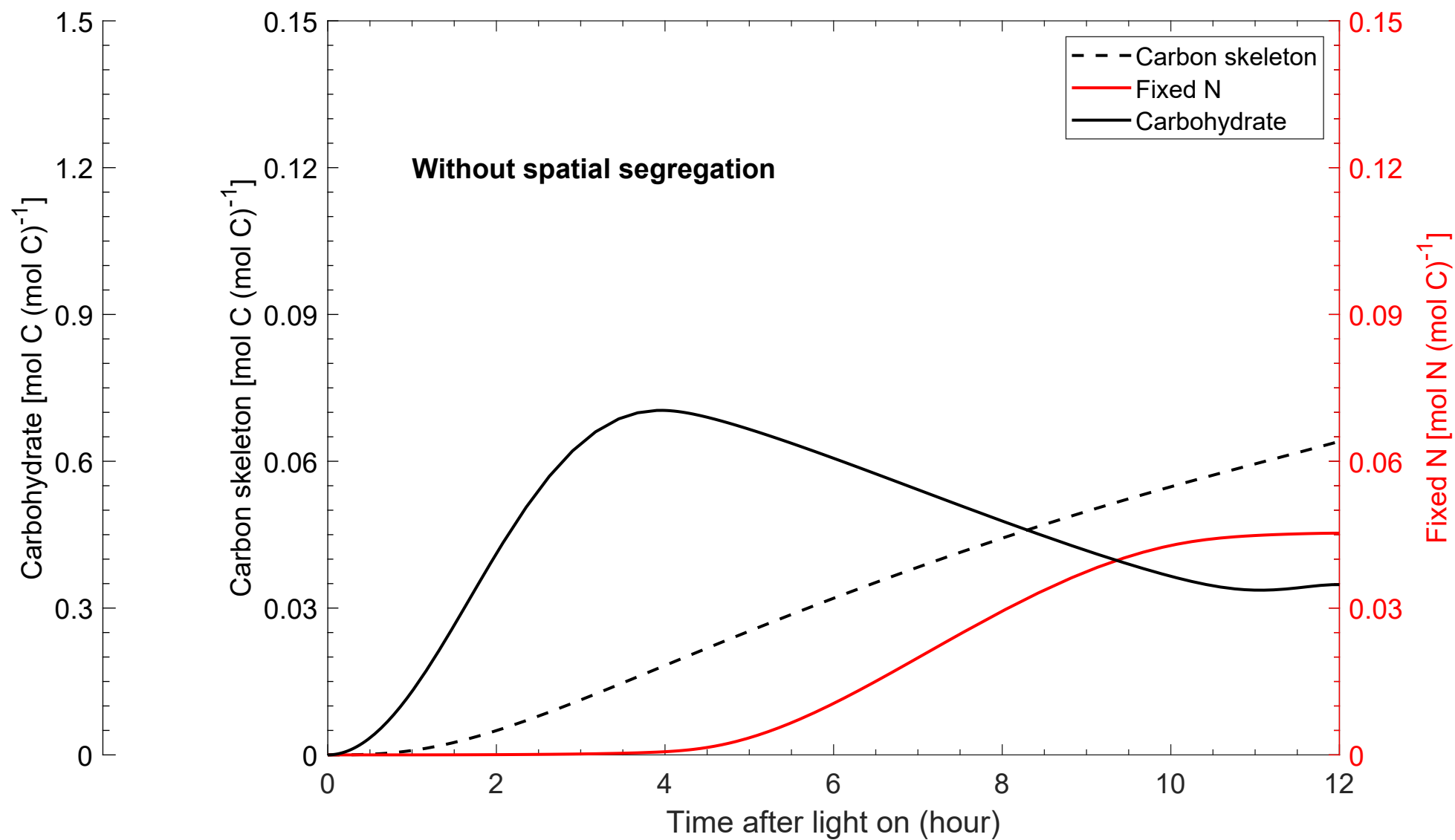

Supplement: FIG S2 [file msystems.00538-22-s0002.pdf]

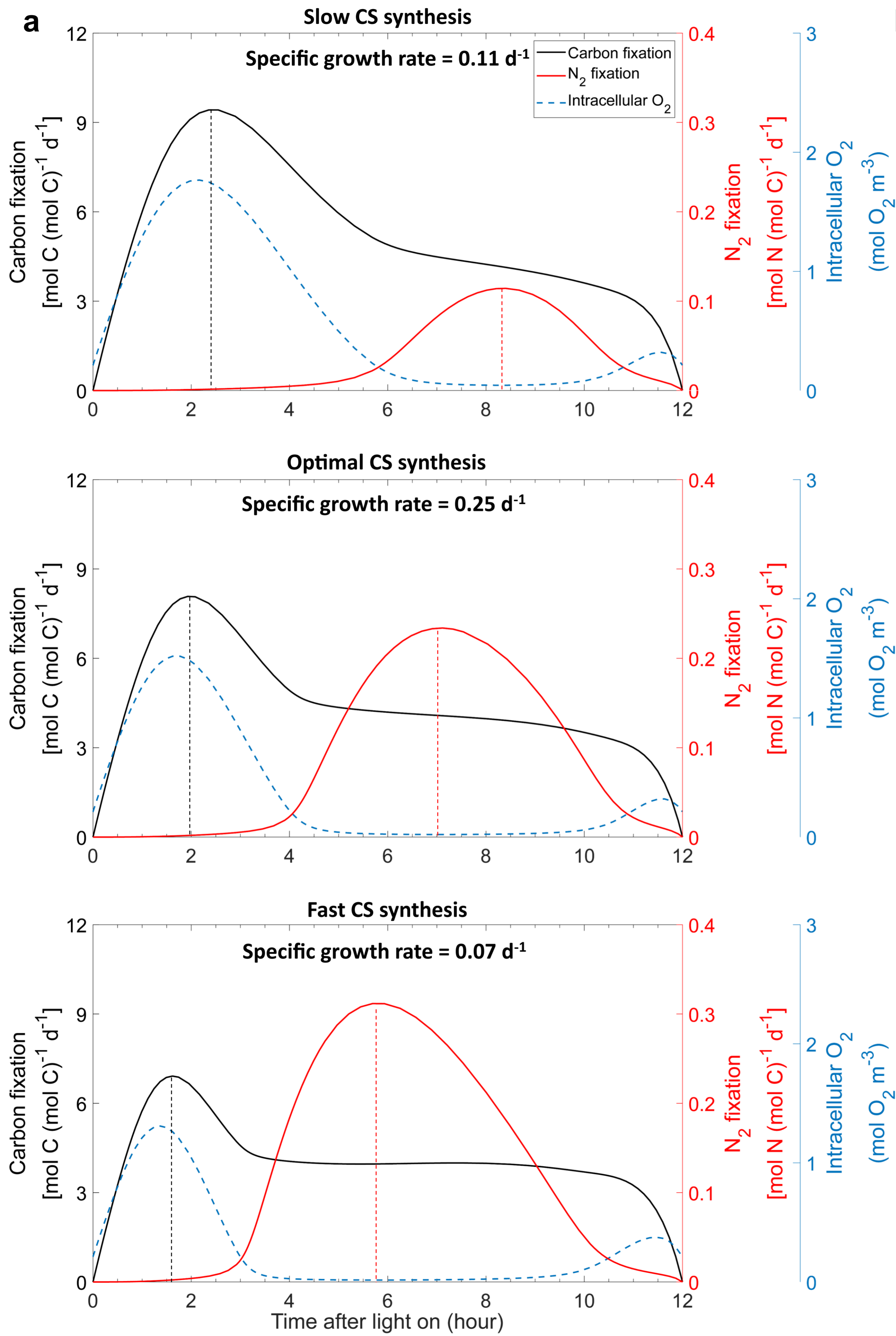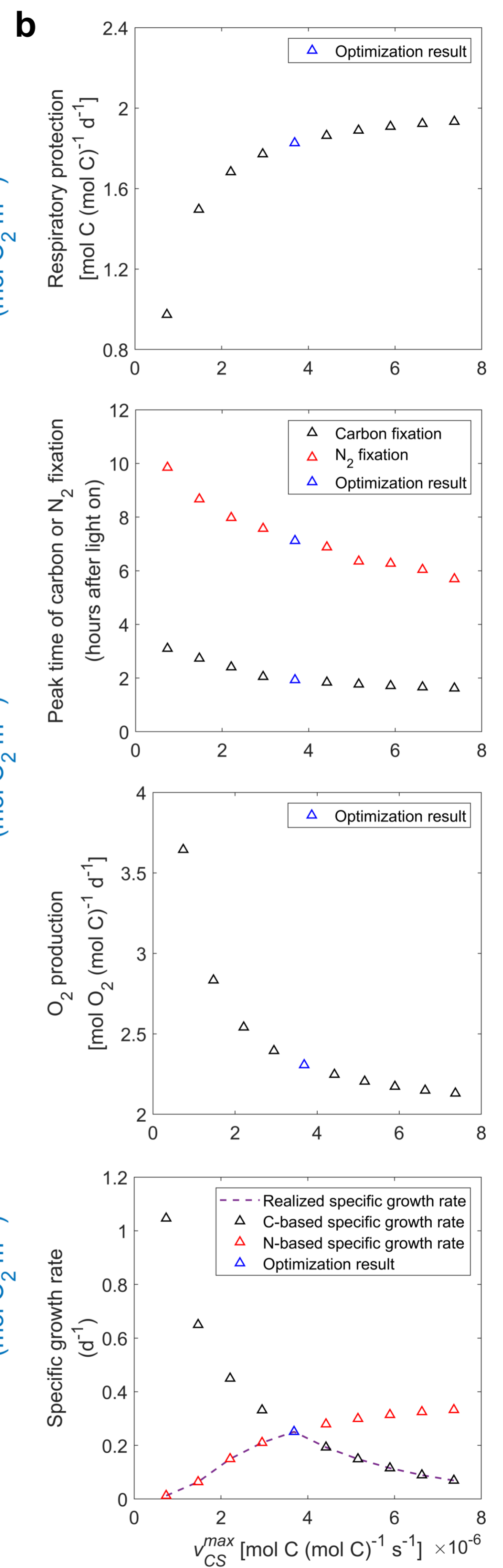

Supplement: FIG S3 [file msystems.00538-22-s0003.pdf]

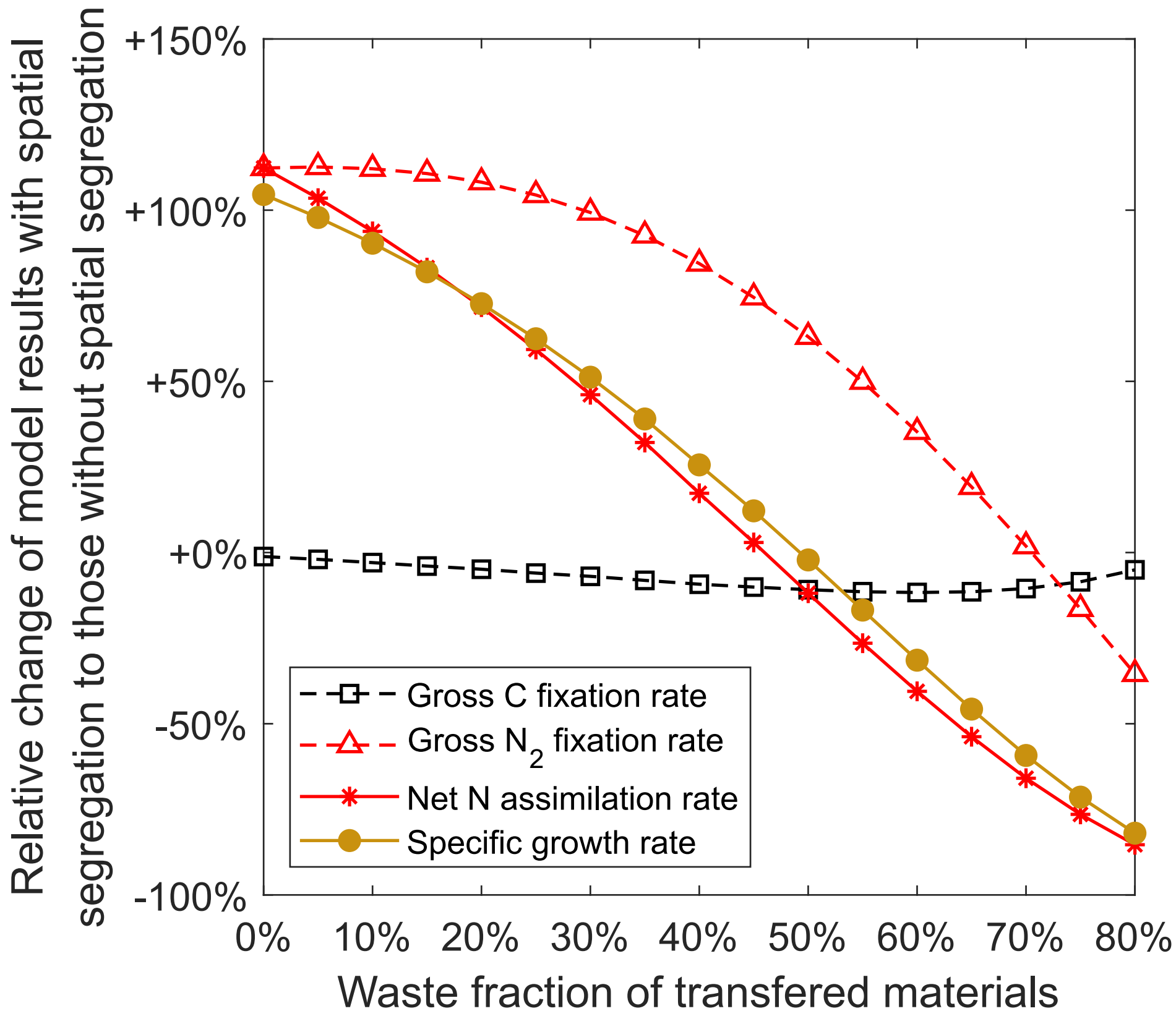

Supplement: FIG S4 [file msystems.00538-22-s0004.pdf]

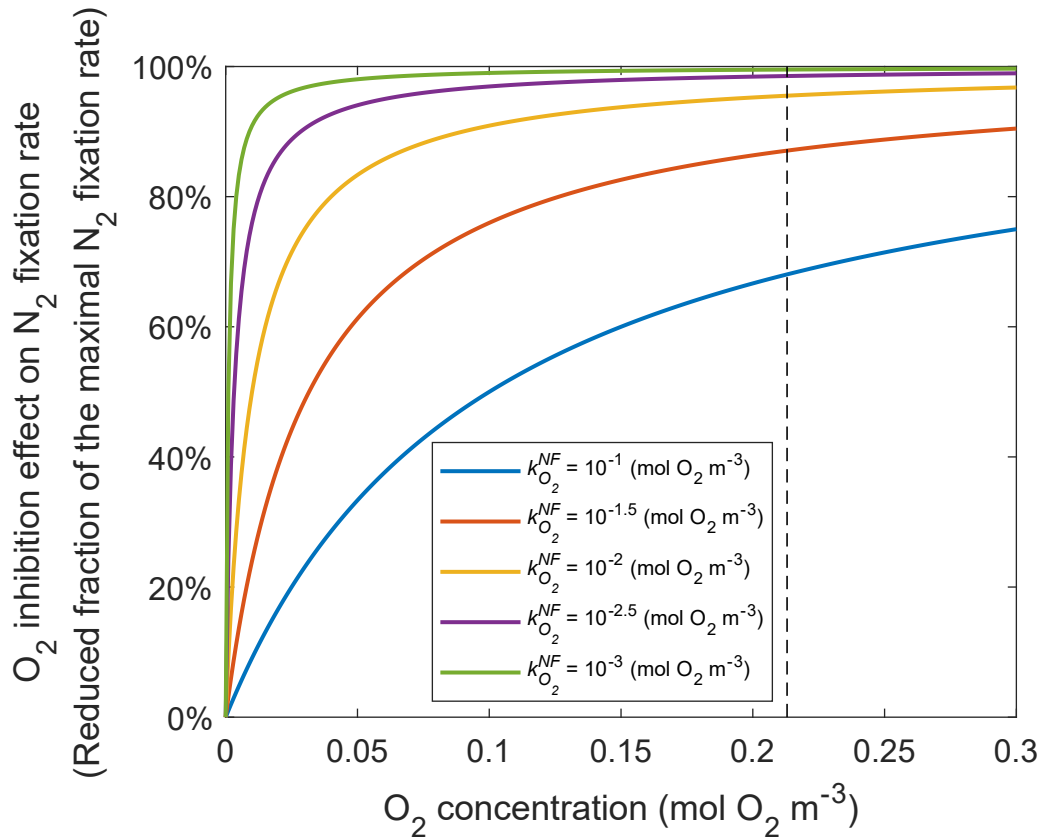

Supplement: FIG S5 [file msystems.00538-22-s0005.pdf]

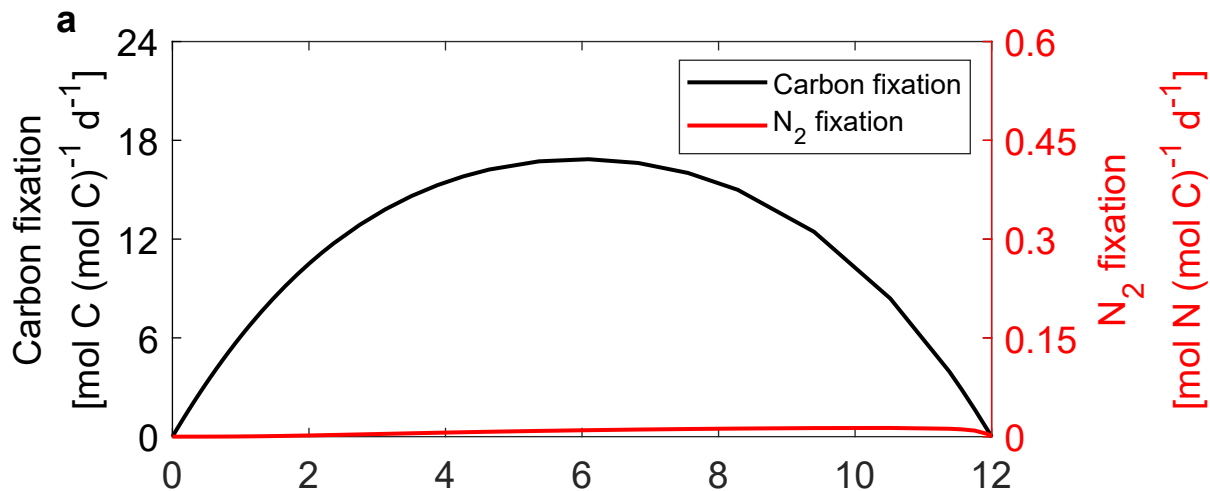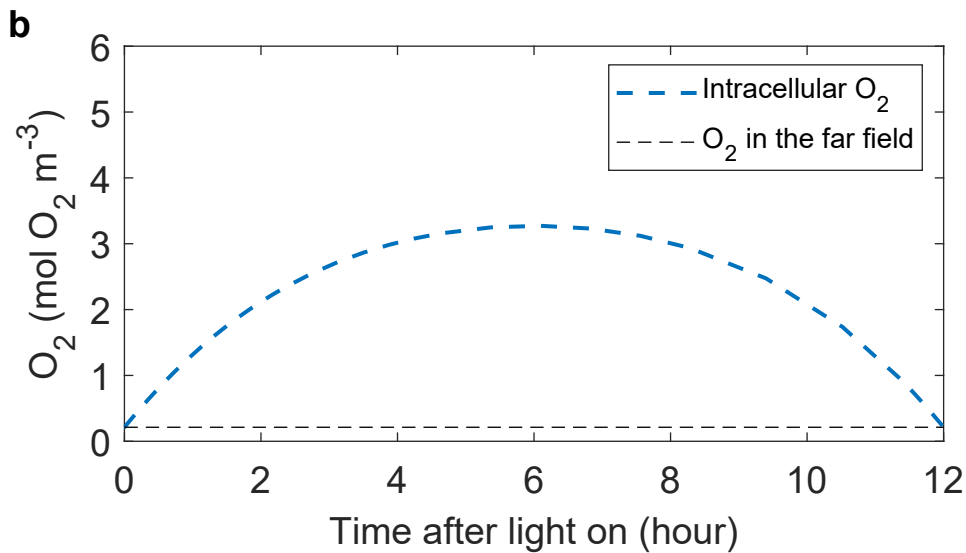

Supplement: FIG S6 [file msystems.00538-22-s0006.pdf]

**a**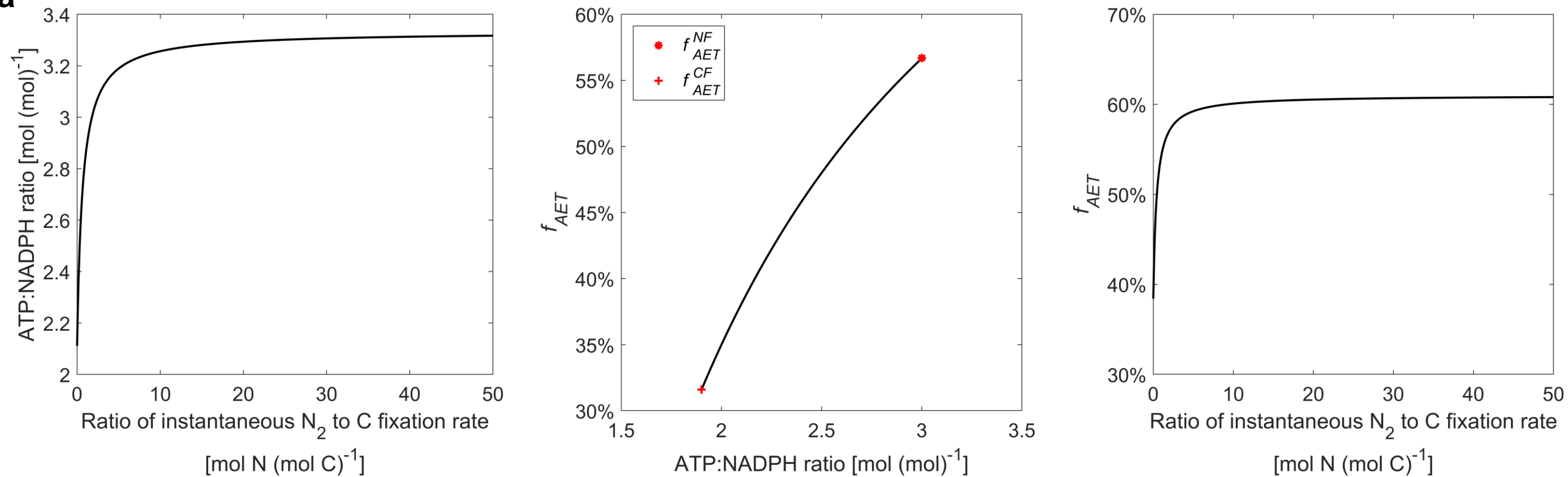**b**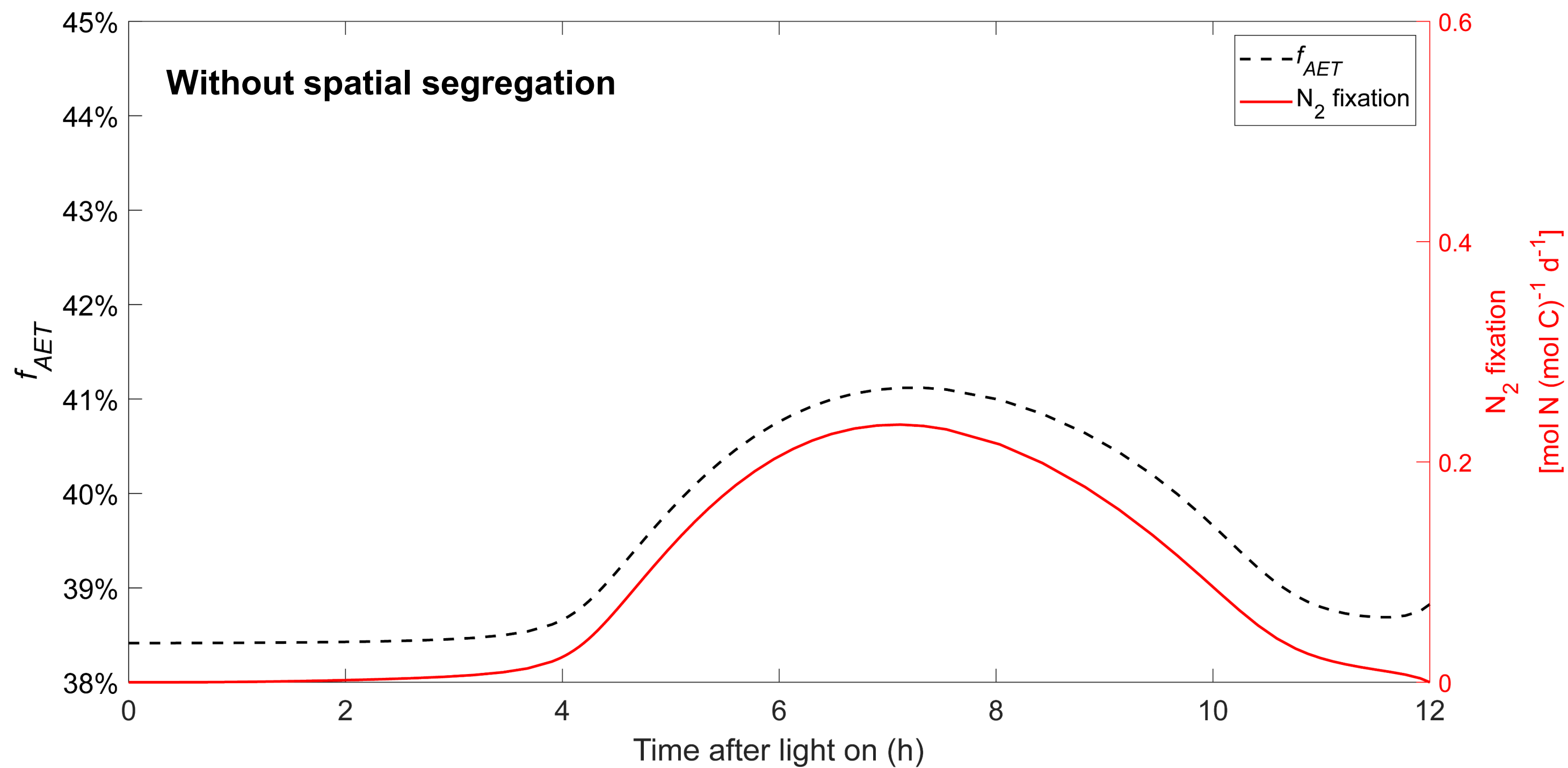

Supplement: FIG S7 [file msystems.00538-22-s0007.pdf]
