# Supplementary material for: N2 Fixation in Trichodesmium Does Not Require Spatial Segregation from Photosynthesis
Source: mSystems. 2022 Jul 11;7(4):e00538-22. doi: 10.1128/msystems.00538-22 (PMC9426587; doi:10.1128/msystems.00538-22)
Supplement: TEXT S1 [file msystems.00538-22-s0010.pdf]

Text S1: Full model description

1. Light intensity and photosynthetic pathways

The daytime light intensity (I, µmol m^{-2} s^{-1}) is (1):

$$I(t) = I_{max} \cdot \sin(\frac{\pi \cdot t}{43,200}), \qquad (S1)$$

where I_{max} is the maximal light intensity of 100 µmol m^{-2} s^{-1} and t is the time (s) during a 12-hour light period.

The light-dependent PET rate [V^{I}_{PET}, mol electron (mol C)$^{-1}$ s^{-1}] is:

$$V^{I}_{PET} = v^{max}_{PET} \cdot (1 - e^{-\alpha_I \cdot I}), \qquad (S2)$$

where v^{max}_{PET} [mol electron (mol C)$^{-1}$ s^{-1}] is the maximal PET rate and α_I (µmol^{-1} m^2 s) is the initial slope of photosynthesis versus light curve.

The realized PET rate [V_{PET}, mol electron (mol C)$^{-1}$ s^{-1}] is:

$$V_{PET} = V^{I}_{PET} \cdot e^{-\beta \cdot V_{RP}}, \qquad (S3)$$

where β [mol C (mol C)$^{-1}$ s] represents the strength of the inhibition effect, and V_{RP} [mol C (mol C)$^{-1}$ s^{-1}] is the RP rate described later.

By absorbing the energy from light, water is decomposed in photosystem II (PSII), with 4 mol of electrons produced for 1 mol of O_2 production. Afterward, electrons are fractionated to LPET and AET to produce NADPH and ATP (2, 3). For 4 electrons through LPET, 2.6 ATP and 2 NADPH are produced, while AET generates 2.6 ATP but no net NADPH and O_2 (2). The overall stoichiometry of LPET and AET is (2):

$$\text{LPET: } 2\ H_2O + 2\ NADP^+ + 2.6\ ADP + 2.6\ Pi + 8\ \text{photons} \rightarrow 2\ NADPH + 2.6\ ATP + 4\ H^+ + O_2, \qquad (S4)$$

$$\text{AET:}\quad 2\,H_2O + 2.6\,ADP + 2.6\,Pi + 8\text{ photons} + O_2 \rightarrow 2.6\,ATP + 4\,H^+ + O_2. \qquad \text{(S5)}$$

AET produces only ATP; therefore, the total NADPH [V_{NADPH}^{PET}, mol NADPH (mol C)$^{-1}$ s^{-1}] and ATP [V_{ATP}^{PET}, mol ATP (mol C)$^{-1}$ s^{-1}] production rates of PET are:

$$V_{NADPH}^{PET} = V_{PET} \cdot (1 - f_{AET}) \cdot q_{LPET}^{NADPH}, \qquad \text{(S6)}$$

$$V_{ATP}^{PET} = V_{PET} \cdot (1 - f_{AET}) \cdot q_{LPET}^{ATP} + V_{PET} \cdot f_{AET} \cdot q_{AET}^{ATP}, \qquad \text{(S7)}$$

where f_{AET} is the fraction of AET and calculated at each time step based on ATP and NADPH requirements (see below), q_{LPET}^{NADPH} = 0.5 mol NADPH (mol electron)$^{-1}$ is the ratio of NADPH production to electrons, and q_{LPET}^{ATP} and q_{AET}^{ATP} = 0.65 mol ATP (mol electron)$^{-1}$ are the ratios of ATP production to electrons in LPET and AET, respectively.

O_2 is produced from LPET [mol O_2 (mol C)$^{-1}$ s^{-1}] at:

$$V_{O_2} = V_{PET} \cdot (1 - f_{AET}) \cdot q_{LPET}^{O_2}, \qquad \text{(S8)}$$

where $q_{LPET}^{O_2}$ = 0.25 mol O_2 (mol electron)$^{-1}$ is O_2 production per electron.

2. N_2 fixation

The maximal potential N_2 fixation rate [V_{NF}^{max}, mol N (mol C)$^{-1}$ s^{-1}] in our model is determined by assuming that the total NADPH and ATP produced by PET are fully utilized by N_2 fixation:

$$V_{NF}^{max} = V_{PET} \cdot (1 - f_{AET}^{NF}) \cdot \frac{q_{LPET}^{NADPH}}{q_{NF}^{NADPH}}, \qquad \text{(S9)}$$

where f_{AET}^{NF} = 56.7% is determined by the ratio of ATP to NADPH [q_{NF}^{NADPH} = 3 mol NADPH (mol N)$^{-1}$] required by N_2 fixation (4, 5).

N_2 fixation in the model also depends on the carbon skeleton [CS, mol C (mol C)$^{-1}$], the already fixed N [N, mol N (mol C)$^{-1}$] and intracellular O_2 [O_2, mol O_2 m^{-3}]:

$$V_{NF} = V_{NF}^{max} \cdot \frac{CS}{CS + k_{CS}^{NF}} \cdot (\frac{N_{max} - N}{N_{max}}) \cdot (1 - \frac{O_2}{O_2 + k_{O_2}^{NF}}), \quad \text{(S10)}$$

where k_{CS}^{NF} [mol C (mol C)$^{-1}$] is the half-saturating coefficient of the carbon skeleton for N_2 fixation. We assume that *Trichodesmium* tends to regulate N_2 fixation down when the fixed N is approaching the maximal N storage [N_{max}, mol N (mol C)$^{-1}$] (6). The modeled O_2 inhibition on N_2 fixation rate is using a Michaelis–Menten equation (7), where the half-saturation coefficient ($k_{O_2}^{NF}$) for the inhibition is unknown and is determined to be 1×10^{-2} mol O_2 m^{-3} by conducting model experiments (see Discussion and Fig. 6) to fit the ratio of modeled gross fixed C to N to an observed value in Finzi-Hart *et al.* (8).

The total NADPH [V_{NADPH}^{NF}, mol NADPH (mol C)$^{-1}$ s^{-1}] and ATP [V_{ATP}^{NF}, mol ATP (mol C)$^{-1}$ s^{-1}] consumption rates in N_2 fixation are:

$$V_{NADPH}^{NF} = V_{NF} \cdot q_{NF}^{NADPH}, \quad \text{(S11)}$$

$$V_{ATP}^{NF} = V_{NF} \cdot q_{NF}^{ATP}, \quad \text{(S12)}$$

where q_{NF}^{ATP} = 9 mol ATP (mol N)$^{-1}$ is the required rate of ATP by N_2 fixation (4, 5).

3. Fraction of AET and carbon fixation

N_2 fixation and carbon fixation require different ratios of ATP to NADPH (3:1 and 1.9:1, respectively; see below). At each time step, after calculating the N_2 fixation rate, the model dynamically adjusts the fraction of AET in PET (f_{AET}), consequently the ratio of produced ATP to NADPH, to fulfill the requirement by N_2 fixation rate and meanwhile to maximize the carbon fixation rate [V_{CF}, mol C (mol C)$^{-1}$ s^{-1}] (Fig. S7a).

The total NADPH consumed by N_2 fixation and carbon fixation is supported by LPET:

$$V_{PET} \cdot (1 - f_{AET}) \cdot q^{NADPH}_{LPET} = V_{NF} \cdot q^{NADPH}_{NF} + V_{CF} \cdot q^{NADPH}_{CF}, \qquad \text{(S13)}$$

where q^{NADPH}_{CF} = 2 mol NADPH (mol C)$^{-1}$ is the required rate of NADPH by carbon fixation (9). The total intracellular ATP requirement includes N_2 fixation, carbon fixation and maintenance, where γ_{MT} = 10% represents an additional ATP requirement by maintenance (10). The ATP requirement is then balanced with its production:

$$V_{PET} \cdot (1 - f_{AET}) \cdot q^{ATP}_{LPET} + V_{PET} \cdot f_{AET} \cdot q^{ATP}_{AET} = (V_{NF} \cdot q^{ATP}_{NF} + V_{CF} \cdot q^{ATP}_{CF}) \cdot (1 + \gamma_{MT}), \qquad \text{(S14)}$$

where q^{ATP}_{CF} = 3.8 mol NADPH (mol C)$^{-1}$ is the required rate of NADPH by carbon fixation, with 3 ATP for producing carbohydrates based on the stoichiometry of the Calvin-Benson cycle (9), and an additional 0.8 ATP for the CO_2 concentrating mechanism by assuming 50% leakage of C_i (inorganic carbon including CO_2 and HCO_3^-), 80% of C_i from HCO_3^-, and a transport cost of 0.5 ATP per HCO_3^- (11, 12).

The two unknown variables in eqns. S13 and S14, f_{AET} and V_{CF}, then can be solved at each time step.

The carbon skeleton in the model is produced from carbohydrates without energy consumption or carbon loss (13). The production rate of the carbon skeleton [V_{CS}, mol C (mol C)$^{-1}$ s^{-1}] is stimulated by the concentration of carbohydrates (CH_2O) [mol C (mol C)$^{-1}$] using a Michaelis–Menten equation (7), and is inhibited by its own accumulation (6):

$$V_{CS} = v^{max}_{CS} \cdot \frac{CH_2O}{CH_2O + k^{CS}_{CH_2O}} \cdot \frac{CS_{max} - CS}{CS_{max}}, \qquad \text{(S15)}$$

where v_{CS}^{max} [mol C (mol C)$^{-1}$ s^{-1}] is the maximal production rate of the carbon skeleton, $k_{CH_2O}^{CS}$ [mol C (mol C)$^{-1}$] is the half-saturation constant of carbohydrates for carbon skeleton production, and CS_{max} [mol C (mol C)$^{-1}$] is the maximum CS storage.

4. Respiratory protection

To create a low-O_2 environment for N_2 fixation, high intracellular O_2 stimulates RP. The rate of RP is also stimulated by the potential of N_2 fixation, which is in turn elevated by light and CS and is limited by fixed N (6, 14). We then parameterize the rate of RP [mol C (mol C)$^{-1}$ s^{-1}] as follows:

$$V_{RP} = v_{RP}^{max} \cdot \frac{O_2}{O_2 + k_{O_2}^{NF}} \cdot (1 - e^{-\alpha_I \cdot I}) \cdot \frac{CS}{CS + k_{CS}^{NF}} \cdot (\frac{N_{max} - N}{N_{max}}), \tag{S16}$$

where v_{RP}^{max} [mol C (mol C)$^{-1}$ s^{-1}] is the maximal RP rate. Note that we use the same half-saturating coefficient of O_2 ($k_{O_2}^{NF}$) in this scheme and that for O_2 inhibition on N_2 fixation (Eq. 3 in the main text). That is, the O_2 inhibition on N_2 fixation stimulates RP to the same degree.

The O_2 consumption rate in RP [$V_{O_2}^{RP}$, mol O_2 (mol C)$^{-1}$ s^{-1}] is:

$$V_{O_2}^{RP} = V_{RP} \cdot q_C^{O_2}, \tag{S17}$$

where $q_C^{O_2}$ [mol O_2 (mol C)$^{-1}$] is the ratio of O_2 to carbon in carbohydrate respiration.

5. O_2 diffusion

O_2 diffusion rate between the cell cytoplasm and ambient environment (T_{O_2}, mol O_2 m^{-3} s^{-1}) is simulated using a scheme by Staal *et al.* (15):

$$T_{O_2} = \frac{-2 \cdot \pi \cdot d_{O_2} \cdot L}{V} \cdot \left\{\frac{1}{\varepsilon} \cdot \ln\left(\frac{R}{R + L_g}\right) - \ln\left(\frac{R + L_g + L_b}{R + L_g}\right)\right\}^{-1} \cdot (O_2^E - O_2), \qquad \text{(S18)}$$

where O_2^E is the ambient far-field O_2 concentration set to a saturating concentration (0.213 mol O_2 m^{-3}) under a typical ocean condition of 34 PSU salinity and 25 °C (16), d_{O_2} (m^2 s^{-1}) is the O_2 diffusion coefficient in seawater, L (m) and V (m^3) are the length and volume of the trichome (simplified to be cylindrical geometry, $V = \pi \cdot (R + L_g)^2 \cdot L$), respectively, ε (dimensionless) is the ratio of the O_2 diffusion coefficient of the cell membrane relative to d_{O_2} and is determined along with $k_{O_2}^{NF}$ by model experiments as mentioned above, R (m) is the radius of the cytoplasm, L_g (m) is the thickness of the cell membrane, and $L_b = 1024 \cdot (R + L_g)$ is the thickness of the boundary layer (17).

6. Integration of state variables during the daytime

The temporal change rates of state variables of carbohydrates, carbon skeleton, fixed N and intracellular O_2 are represented in ordinary differential equations (ODEs), including all the fluxes described above. Note that from the model design, NADPH and ATP are not stored but are fully consumed at each time step. Basically, all the rates described above are normalized to carbon biomass. An exception is for the ODE of O_2, because it is in a unit volumetric concentration (mol O_2 m^{-3}):

$$\frac{dCH_2O}{dt} = V_{CF} - V_{CS} - V_{RP}, \qquad \text{(S19)}$$

$$\frac{dCS}{dt} = V_{CS}, \qquad \text{(S20)}$$

$$\frac{dN}{dt} = V_{NF}, \qquad \text{(S21)}$$

$$\frac{dO_2}{dt} = (V_{O_2} - V_{O_2}^{RP}) \cdot Q_C + T_{O_2}, \qquad \text{(S22)}$$

where Q_C = 18333 mol C m^{-3} is the cellular carbon biomass quota (18). These ODEs are integrated over the light period (12 hours) using the MATLAB ode15s integrator (19).

7. Biosynthesis and growth rate

Trichodesmium might store newly fixed C and N during the daytime and assimilate them into biomass, mainly during the dark period (8). Therefore, for simplification, the model calculates the amount of biomass [*Bio*, mol C (mol C)$^{-1}$] that can be synthesized using the carbohydrates, carbon skeletons and fixed N at the end of the light period. *Bio* is the smaller of N-based (Bio_N) and C-based biomass (Bio_C), with Bio_N calculated by dividing fixed N to the molar N:C (0.159) (20). Bio_C is calculated from the carbohydrates and carbon skeleton considering mass and energy balance. The energy needed for biosynthesis is from the respiration of carbohydrates ($CH_2O_{BIO}^{RESP}$):

$$Bio_C \cdot q_{BIO}^{ATP} \cdot (1 + \gamma_{MT}) = CH_2O_{BIO}^{RESP} \cdot q_{RESP}^{ATP}, \qquad \text{(S23)}$$

where q_{BIO}^{ATP} = 2 mol ATP (mol C)$^{-1}$ is the ATP requirement rate by biosynthesis (6), and q_{RESP}^{ATP} = 5 mol ATP (mol C)$^{-1}$ is the ATP production rate from respiring carbohydrates (21). Meanwhile, the non-respired carbohydrates and all the carbon skeletons are involved in biosynthesis:

$$Bio_C = CH_2O - CH_2O_{BIO}^{RESP} + CS. \qquad \text{(S24)}$$

Bio_C then can be solved from the above two equations. Note that the carbohydrate respiration calculated in this step is counted in the daily integrated respiration.

Noting that all the rates have been normalized to carbon biomass, *Bio* is therefore the relative increase in biomass over one day. The growth rate (*G*) is then the natural log of (1 + *Bio*) divided by 1 day.

8. Model design of spatial segregation

The single-trichome model introduced above is then modified to represent the N_2-fixing (diazocytes) and the photosynthetic cells by incorporating the spatial segregation between photosynthesis and N_2 fixation. The fraction (f_D) of the trichome cells assigned to diazocytes is set to 15% (22, 23), and the rest are photosynthetic cells. It is assumed in the model that all the materials except O_2 are actively and quickly transferred between photosynthetic cells and diazocytes, and hence distribute evenly along the trichome (8). Therefore, these variables are not simulated separately for photosynthetic cells and diazocytes, but their total fluxes are calculated according to the fractions of these two types of cells.

PET is confined to photosynthetic cells, and its rate [V^P_{PET}, mol electron (mol C)$^{-1}$ s^{-1}] is:

$$V^P_{PET} = V^I_{PET} \cdot e^{-\beta \cdot V^P_{RP}}, \qquad \text{(S25)}$$

$$V_{PET} = V^P_{PET} \cdot (1 - f_D), \qquad \text{(S26)}$$

where V^P_{RP} [mol C (mol C)$^{-1}$ s^{-1}], described later, is the RP rate in photosynthetic cells, and V_{PET} [mol electron (mol C)$^{-1}$ s^{-1}] is the PET rate in the whole trichome.

The total NADPH and ATP production rates of PET in the whole trichome are calculated as shown in eqns. S6 and S7. No net O_2 is produced in diazocytes, while O_2 is produced from LPET [mol O_2 (mol C)$^{-1}$ s^{-1}] in photosynthetic cells at:

$$V_{O_2^P} = V^P_{PET} \cdot (1 - f_{AET}) \cdot q^{O_2}_{LPET}. \qquad \text{(S27)}$$

N_2 fixation in the model with spatial segregation only occurs in diazocytes, and the calculation of its whole trichome specific rate is already described in eqn. S10, with the O_2 concentration in

diazocytes, while CO_2 concentrating mechanism (CCM) and carbon fixation only occur in photosynthetic cells, and the whole trichome specific carbon fixation rate is determined as that in the model without spatial segregation. Carbon skeleton production and biosynthesis occur in the whole trichome, and their rates are also computed as those in the model without spatial segregation.

However, intracellular O_2 in photosynthetic cells (O_2^P, mol O_2 m^{-3}) and in diazocytes (O_2^D, mol O_2 m^{-3}) is simulated separately. Respiratory protection rates are calculated as follows:

$$V^{RP}_{O_2^P} = v^{max}_{RP} \cdot \frac{O_2^P}{O_2^P + k^{NF}_{O_2}} \cdot (1 - e^{-\alpha_I \cdot I}) \cdot \frac{CS}{CS + k^{NF}_{CS}} \cdot (\frac{N_{max} - N}{N_{max}}) \cdot q_C^{O_2}, \quad \text{(S28)}$$

$$V^{RP}_{O_2^D} = v^{max}_{RP} \cdot \frac{O_2^D}{O_2^D + k^{NF}_{O_2}} \cdot (1 - e^{-\alpha_I \cdot I}) \cdot \frac{CS}{CS + k^{NF}_{CS}} \cdot (\frac{N_{max} - N}{N_{max}}) \cdot q_C^{O_2}, \quad \text{(S29)}$$

A mixed layer of O_2 with a thickness equivalent to the cell radius ($L_m = R + L_g$) is assumed to form around the surface of the trichome (Fig. S1). The rates of O_2 diffusion occurring at different interfaces are parameterized by adopting the scheme of Staal *et al.* (15) considering the fractions of photosynthetic cells and diazocytes:

$$T^E_M = \frac{-2 \cdot \pi \cdot d_{O_2} \cdot L}{V} \cdot \left\{ \ln\left(\frac{R + L_g + L_m}{R + L_g + L_m + L_b} \right) \right\}^{-1} \cdot (O_2^E - O_2^M), \quad \text{(S30)}$$

$$T^M_P = \frac{-2 \cdot \pi \cdot d_{O_2} \cdot L_P}{V} \cdot \left\{ \frac{1}{\varepsilon} \cdot \ln\left(\frac{R}{R + L_g} \right) \right\}^{-1} \cdot (O_2^M - O_2^P), \quad \text{(S31)}$$

$$T^M_D = \frac{-2 \cdot \pi \cdot d_{O_2} \cdot L_D}{V} \cdot \left\{ \frac{1}{\varepsilon} \cdot \ln\left(\frac{R}{R + L_g} \right) \right\}^{-1} \cdot (O_2^M - O_2^D), \quad \text{(S32)}$$

$$T^P_D = \frac{2 \cdot \pi \cdot R^2 \cdot d_{O_2} \cdot \varepsilon}{V \cdot L_g} \cdot (O_2^P - O_2^D), \quad \text{(S33)}$$

where *E*, *M*, *P* and *D* appearing in the superscripts or subscripts represent the ambient environment, the mixed layer, photosynthetic cells and diazocytes, respectively; *T* represents the O_2 diffusion from the pool denoted by its superscript to that by the subscript; $L_P = (1 - f_D) \cdot L$ and $L_D = f_D \cdot L$ are the total length of photosynthetic cells and diazocytes, respectively; L, L_g and L_b are already described in eqn. S18. O_2 exchange between the photosynthetic cells and the diazocytes is much slower, due to the small area of the interface, than that between the environment and these cells.

The change rates of carbohydrates, carbon skeleton and fixed N are already shown in eqns. S19 to S21, and those of the oxygen state variables are:

$$\frac{dO_2^M}{dt} = (T_M^E - T_P^M - T_D^M) \cdot \frac{V}{V_m}, \tag{S34}$$

$$\frac{dO_2^P}{dt} = (V_{O_2^P} - V_{O_2^P}^{RP}) \cdot Q_C + (T_P^M - T_D^P) \cdot \frac{V}{V_P}, \tag{S35}$$

$$\frac{dO_2^D}{dt} = - V_{O_2^D}^{RP} \cdot Q_C + (T_D^M + T_D^P) \cdot \frac{V}{V_D}, \tag{S36}$$

where V_M, V_P and V_D (m^3) are the volume of the mixed layer, photosynthetic cells and diazocytes, respectively.

<u>9. Model experiments of the degree of temporal segregation</u>

In the model, a series of processes determines the degree of temporal segregation between photosynthesis and N_2 fixation and impacts the growth rate. To better evaluate the effect, we perform model experiments by varying the parameter controlling the synthesis rate of the carbon skeletons (Fig. S3a). A halved synthesis rate of the carbon skeletons (Fig. S3a) slows the increase in N_2 fixation potential and weakens RP (Fig. S3b). PET is in turn inhibited less, the period of increasing carbon fixation is maintained longer, and more organic carbon and O_2 are produced (Fig. S3). However, the low-O_2 window appears much later and shorter because of

both higher O_2 production and lower RP (Fig. S3). Therefore, the lowered N_2 fixation reduces the modeled growth rate by ~60% even when the carbon fixation is higher (Fig. S3). Under this circumstance, the temporal segregation between photosynthesis and N_2 fixation is wider (Fig. S3a). In comparison, a doubled synthesis rate of the carbon skeletons leads to a higher and earlier peak of N_2 fixation and a narrower temporal segregation (Fig. S3). However, less carbon fixation and a higher RP cause the modeled *Trichodesmium* to grow under carbon limitation, which also reduced the growth rate by ~70% (Fig. S3b). Note that in both experiments, *Trichodesmium* grows inefficiently with either fixed carbon or N being partly wasted, which however, does not occur in the optimized case (Fig. S3). Our model suggests a possible mechanism of feedback that *Trichodesmium* uses to ensure temporal segregation between its photosynthesis and N_2 fixation, while a proper degree of the temporal segregation can be a key to ensuring the organism's growth efficiently (Fig. S3a).

Supplementary References

1. Reimers AM, Knoop H, Bockmayr A, Steuer R. 2017. Cellular trade-offs and optimal resource allocation during cyanobacterial diurnal growth. Proc Natl Acad Sci USA 114:E6457-E6465.
2. Geider RJ, Moore CM, Ross ON. 2009. The role of cost–benefit analysis in models of phytoplankton growth and acclimation. Plant Ecol Div 2:165-178.
3. Allen JF. 2003. Cyclic, pseudocyclic and noncyclic photophosphorylation: New links in the chain. Trends Plant Sci 8:15-19.
4. Flores E, Herrero A. 1994. Assimilatory nitrogen metabolism and its regulation, p 487-517. *In* Bryant DA (ed), The Molecular Biology of Cyanobacteria. Kluwer Academic Publishers, Dordrecht.
5. Flores E, Frías JE, Rubio LM, Herrero A. 2005. Photosynthetic nitrate assimilation in cyanobacteria. Photosynth Res 83:117-133.
6. Inomura K, Wilson ST, Deutsch C. 2019. Mechanistic model for the coexistence of nitrogen fixation and photosynthesis in marine *Trichodesmium*. mSystems 4:e00210-19.
7. Rogers A, Gibon Y. 2009. Enzyme kinetics: Theory and practice, p 71-103. *In* Schwender J (ed), Plant Metabolic Networks doi:10.1007/978-0-387-78745-9_4. Springer, New York.
8. Finzi-Hart JA, Pett-Ridge J, Weber PK, Popa R, Fallon SJ, Gunderson T, Hutcheon ID, Nealson KH, Capone DG. 2009. Fixation and fate of C and N in the cyanobacterium *Trichodesmium* using nanometer-scale secondary ion mass spectrometry. Proc Natl Acad Sci USA 106:6345-6350.
9. Baker NR, Harbinson J, Kramer DM. 2007. Determining the limitations and regulation of photosynthetic energy transduction in leaves. Plant Cell Environ 30:1107-1125.
10. Luo YW, Shi D, Kranz SA, Hopkinson BM, Hong H, Shen R, Zhang F. 2019. Reduced nitrogenase efficiency dominates response of the globally important nitrogen fixer *Trichodesmium* to ocean acidification. Nat Commun 10:1521.
11. Eichner M, Thoms S, Kranz SA, Rost B. 2015. Cellular inorganic carbon fluxes in *Trichodesmium*: A combined approach using measurements and modelling. J Exp Bot 66:749-759.
12. Raven JA, Beardall J, Giordano M. 2014. Energy costs of carbon dioxide concentrating mechanisms in aquatic organisms. Photosynth Res 121:111-124.
13. Rabouille S, Staal M, Stal LJ, Soetaert K. 2006. Modeling the dynamic regulation of nitrogen fixation in the cyanobacterium *Trichodesmium* sp. Appl Environ Microbiol 72:3217-27.
14. Inomura K, Deutsch C, Wilson ST, Masuda T, Lawrenz E, Lenka B, Sobotka R, Gauglitz JM, Saito MA, Prášil O, Follows MJ. 2019. Quantifying oxygen management and temperature and light dependencies of nitrogen fixation by *Crocosphaera watsonii*. mSphere 4:e00531-19.
15. Staal M, Meysman FJ, Stal LJ. 2003. Temperature excludes N_2-fixing heterocystous cyanobacteria in the tropical oceans. Nature 425:504-507.
16. Benson BB, Krause D. 1984. The concentration and isotopic fractionation of oxygen dissolved in freshwater and seawater in equilibrium with the atmosphere. Limnol Oceanogr 29:620-632.
17. Klawonn I, Eichner MJ, Wilson ST, Moradi N, Thamdrup B, Kummel S, Gehre M, Khalili A, Grossart HP, Karl DM, Ploug H. 2020. Distinct nitrogen cycling and steep chemical gradients in *Trichodesmium* colonies. ISME J 14:399-412.

18. Bratbak G, Dundas I. 1984. Bacterial dry matter content and biomass estimations. Appl Environ Microbiol 48:755-757.
19. Shampine LF, Reichelt MW. 1997. The MATLAB ODE Suite. SIAM J Sci Comput 18:1-22.
20. LaRoche J, Breitbarth E. 2005. Importance of the diazotrophs as a source of new nitrogen in the ocean. J Sea Res 53:67-91.
21. Mitchell P. 1970. Aspects of the chemiosmotic hypothesis. Biochem J 116:5-6.
22. El-Shehawy R, Lugomela C, Ernst A, Bergman B. 2003. Diurnal expression of *hetR* and diazocyte development in the filamentous non-heterocystous cyanobacterium *Trichodesmium erythraeum*. Microbiology 149:1139-1146.
23. Berman-Frank I, Lundgren P, Falkowski P. 2003. Nitrogen fixation and photosynthetic oxygen evolution in cyanobacteria. Res Microbiol 154:157-164.
24. Carpenter EJ, Oneil JM, Dawson R, Capone DG, Siddiqui PJA, Roenneberg T, Bergman B. 1993. The tropical diazotrophic phytoplankter *Trichodesmium*: Biological characteristics of two common species. Mar Ecol Prog Ser 95:295-304.
