# Supplementary material for: N2 Fixation in Trichodesmium Does Not Require Spatial Segregation from Photosynthesis
Source: mSystems. 2022 Jul 11;7(4):e00538-22. doi: 10.1128/msystems.00538-22 (PMC9426587; doi:10.1128/msystems.00538-22)
Supplement: TABLE S1 [file msystems.00538-22-s0008.pdf]

Symbol	Unit	Definition	Value	Source or Note
v_{PET}^{max}	mol electron (mol C)$^{-1}$ s^{-1}	Maximal PET	2.0×10^{-3}	This study[a]
β	(mol C)$^{-1}$ mol C s	Parameter of inhibition effect of respiration on PET	2×10^{4}	This study[a]
N_{max}	mol N (mol C)$^{-1}$	Maximal fixed storage	0.159	This study[b]
CS_{max}	mol C (mol C)$^{-1}$	Maximal carbon skeleton storage	1	This study[c]
$k_{O_2}^{NF}$	mol O_2 m^{-3}	Half-saturating coefficient of O_2 for N_2 fixation	0.01	This study[d]
ε	dimensionless	Relative diffusivity of cell membrane	10^{-4}	This study[d]
α_I	μmol^{-1} m^{2} s	The initial slope of *P* versus *I* curve	0.01	(1)
γ_{MT}	dimensionless	Ratio of the energy consumed by maintenance to all of that by other processes	10%	(2)
d_{O_2}	m^{2} s^{-1}	O_2 diffusion coefficient at 34 PSU and 25 °C	2.26×10^{-9}	(3)
f_D	dimensionless	Fraction of diazocytes in the whole trichome	15%	(4)
		Boundary conditions		
I_{max}	μmol m^{-2} s^{-1}	Maximal light intensity	100	
O_2^E	mol O_2 m^{-3}	Ambient far-field O_2	0.213	
		Elemental or energy stoichiometries of metabolic activities		
q_{LPET}^{NADPH}	mol NADPH (mol electron)$^{-1}$	NADPH/electron ratio of LPET	0.5	(5)
q_{LPET}^{ATP}	mol ATP (mol electron)$^{-1}$	ATP/electron ratio of LPET	0.65	(6)
q_{AET}^{ATP}	mol ATP (mol electron)$^{-1}$	ATP/electron ratio of AET	0.65	(6)
$q_{LPET}^{O_2}$	mol O_2 (mol electron)$^{-1}$	O_2/electron ratio of LPET	0.25	(5)
q_{NF}^{NADPH}	mol NADPH (mol N)$^{-1}$	NADPH/N ratio of N_2 fixation	3	(7, 8)
q_{NF}^{ATP}	mol ATP (mol N)$^{-1}$	ATP/N ratio of N_2 fixation	9	(7, 8)
q_{CF}^{NADPH}	mol NADPH (mol C)$^{-1}$	NADPH/C ratio of C fixation	2	(9)
q_{CF}^{ATP}	mol ATP (mol C)$^{-1}$	ATP/C ratio of C fixation including CCM	3.8	(9, 10)
$q_C^{O_2}$	mol O_2 (mol C)$^{-1}$	O_2/C ratio of respiration	1	(11)
q_{BIO}^{ATP}	mol ATP (mol C)$^{-1}$	ATP/C ratio of biosynthesis	2	(1)
q_{RESP}^{ATP}	mol ATP (mol C)$^{-1}$	ATP/C ratio of respiration	5	(11)
f_{AET}^{NF}	dimensionless	The least f_{AET} to support N_2 fixation	56.7%	(6)
Q_C	mol C m^{-3}	Cellular carbon biomass concentration	18333	(12)
		Morphological parameters of Trichodesmium		

L	m	Length of the total trichome	554×10^{-6}	(13)
R	m	Radius of the cytoplasm	4.80×10^{-6}	(13)
L_g	m	Thickness of cell membrane layer	0.076	(13)

[a] Predefined.

[b] By multiplying the initial C biomass with the molar N:C (0.159) of *Trichodesmium* (14).

[c] CS_{max} is set to be the same as the initial C biomass.

[d] $k_{O_2}^{NF}$ and ε are from model experiments (see Discussion and Fig. 6).

LPET: linear photosynthetic electron transfer; AET: alternative electron transfer; CCM: CO_2 concentrating mechanism.

Supplementary References

1. Inomura K, Wilson ST, Deutsch C. 2019. Mechanistic model for the coexistence of nitrogen fixation and photosynthesis in marine *Trichodesmium*. mSystems 4:e00210-19.
2. Luo YW, Shi D, Kranz SA, Hopkinson BM, Hong H, Shen R, Zhang F. 2019. Reduced nitrogenase efficiency dominates response of the globally important nitrogen fixer *Trichodesmium* to ocean acidification. Nat Commun 10:1521.
3. Benson BB, Krause D. 1984. The concentration and isotopic fractionation of oxygen dissolved in freshwater and seawater in equilibrium with the atmosphere. Limnol Oceanogr 29:620-632.
4. El-Shehawy R, Lugomela C, Ernst A, Bergman B. 2003. Diurnal expression of *hetR* and diazocyte development in the filamentous non-heterocystous cyanobacterium *Trichodesmium erythraeum*. Microbiology 149:1139-1146.
5. Allen JF. 2003. Cyclic, pseudocyclic and noncyclic photophosphorylation: New links in the chain. Trends Plant Sci 8:15-19.
6. Geider RJ, Moore CM, Ross ON. 2009. The role of cost–benefit analysis in models of phytoplankton growth and acclimation. Plant Ecol Div 2:165-178.
7. Flores E, Herrero A. 1994. Assimilatory nitrogen metabolism and its regulation, p 487-517. *In* Bryant DA (ed), The Molecular Biology of Cyanobacteria. Kluwer Academic Publishers, Dordrecht.
8. Flores E, Frías JE, Rubio LM, Herrero A. 2005. Photosynthetic nitrate assimilation in cyanobacteria. Photosynth Res 83:117-133.
9. Baker NR, Harbinson J, Kramer DM. 2007. Determining the limitations and regulation of photosynthetic energy transduction in leaves. Plant Cell Environ 30:1107-1125.
10. Raven JA, Beardall J, Giordano M. 2014. Energy costs of carbon dioxide concentrating mechanisms in aquatic organisms. Photosynth Res 121:111-124.
11. Mitchell P. 1970. Aspects of the chemiosmotic hypothesis. Biochem J 116:5-6.
12. Bratbak G, Dundas I. 1984. Bacterial dry matter content and biomass estimations. Appl Environ Microbiol 48:755-757.
13. Carpenter EJ, Oneil JM, Dawson R, Capone DG, Siddiqui PJA, Roenneberg T, Bergman B. 1993. The tropical diazotrophic phytoplankter *Trichodesmium*: Biological characteristics of two common species. Mar Ecol Prog Ser 95:295-304.
14. LaRoche J, Breitbarth E. 2005. Importance of the diazotrophs as a source of new nitrogen in the ocean. J Sea Res 53:67-91.
