# Supplementary material for: N2 Fixation in Trichodesmium Does Not Require Spatial Segregation from Photosynthesis
Source: mSystems. 2022 Jul 11;7(4):e00538-22. doi: 10.1128/msystems.00538-22 (PMC9426587; doi:10.1128/msystems.00538-22)
Supplement: TABLE S2 [file msystems.00538-22-s0009.pdf]

Symbol	Unit	Definition
**State variables**		
CH_2O	mol C (mol C)$^{-1}$	Carbohydrate
CS	mol C (mol C)$^{-1}$	Carbon skeleton
N	mol N (mol C)$^{-1}$	Fixed N
O_2	mol O_2 m^{-3}	Intracellular O_2
**Intermediate process variables**		
I	μmol m^{-2} s^{-1}	Light intensity
t	s	Time
V_{PET}^{I}	mol electron (mol C)$^{-1}$ s^{-1}	Light-dependent PET rate
V_{PET}	mol electron (mol C)$^{-1}$ s^{-1}	PET rate
V_{NADPH}^{PET}	mol NADPH (mol C)$^{-1}$ s^{-1}	NADPH production rate of PET
V_{ATP}^{PET}	mol ATP (mol C)$^{-1}$ s^{-1}	ATP production rate of PET
f_{AET}	dimensionless	Fraction of total electrons in PET to AET
V_{O_2}	mol O_2 (mol C)$^{-1}$ s^{-1}	Net O_2 production rate of PET
V_{NF}^{max}	mol N (mol C)$^{-1}$ s^{-1}	Maximal N_2 fixation rate
V_{NF}	mol N (mol C)$^{-1}$ s^{-1}	N_2 fixation rate
V_{NADPH}^{NF}	mol NADPH (mol C)$^{-1}$ s^{-1}	NADPH consumption rate in N_2 fixation
V_{ATP}^{NF}	mol ATP (mol C)$^{-1}$ s^{-1}	ATP consumption rate in N_2 fixation
V_{CF}	mol C (mol C)$^{-1}$ s^{-1}	Carbon fixation rate
V_{ATP}^{CF}	mol ATP (mol C)$^{-1}$ s^{-1}	ATP consumption rate in carbon fixation
V_{NADPH}^{CF}	mol NADPH (mol C)$^{-1}$ s^{-1}	NADPH consumption rate in carbon fixation
V_{CS}	mol C (mol C)$^{-1}$ s^{-1}	Carbon skeleton production rate
V_{RP}	mol C (mol C)$^{-1}$ s^{-1}	Respiratory protection rate
$V_{O_2}^{RP}$	mol O_2 (mol C)$^{-1}$ s^{-1}	O_2 consumption rate in respiratory protection
T_{O_2}	mol O_2 m^{-3} s^{-1}	O_2 diffusion rate between the cytoplasm and ambient environment
Bio	mol C (mol C)$^{-1}$	New synthesized biomass
Bio_N	mol C (mol C)$^{-1}$	New synthesized N-based biomass
Bio_C	mol C (mol C)$^{-1}$	New synthesized C-based biomass
$CH_2O_{BIO}^{RESP}$	mol C (mol C)$^{-1}$	Respired carbohydrates to fulfill the energy need for biosynthesis
G	d^{-1}	Specific growth rate

Additional model variables for spatial segregation

**State variables**		
O_2^D	mol O_2 m^{-3}	O_2 in photosynthetic cells
O_2^P	mol O_2 m^{-3}	O_2 in diazocytes
O_2^M	mol O_2 m^{-3}	O_2 in the mixed layer
**Intermediate process variables**		
V_{PET}^P	mol electron (mol C)$^{-1}$ s^{-1}	PET rate in photosynthetic cells
$V_{O_2^P}$	mol O_2 (mol C)$^{-1}$ s^{-1}	Net O_2 production rate of PET in photosynthetic cells
$V_{O_2^P}^{RP}$	mol O_2 (mol C)$^{-1}$ s^{-1}	O_2 consumption rate of respiratory protection in photosynthetic cells
$V_{O_2^D}^{RP}$	mol O_2 (mol C)$^{-1}$ s^{-1}	O_2 consumption rate of respiratory protection in diazocytes
T_M^E	mol O_2 m^{-3} s^{-1}	O_2 diffusion rate between the ambient environment and the mixed layer
T_P^M	mol O_2 m^{-3} s^{-1}	O_2 diffusion rate between the mixed layer and photosynthetic cells
T_D^M	mol O_2 m^{-3} s^{-1}	O_2 diffusion rate between the mixed layer and diazocytes
T_D^P	mol O_2 m^{-3} s^{-1}	O_2 diffusion rate between photosynthetic cells and diazocytes

Note: The initial values (t = 0) of CH_2O, *CS* and *N* are set to be 0, and initial O_2 concentration is the same as that of ambient O_2 (0.213 mol O_2 m^{-3}).
